# DepRescribing inapprOpriate Proton Pump InhibiTors (DROPIT): study protocol of a cluster-randomised controlled trial in Swiss primary care

**DOI:** 10.1136/bmjopen-2024-094495

**Published:** 2025-01-20

**Authors:** Angela Edith Schulthess-Lisibach, Renata Vidonscky Lüthold, Clémentine Tombez, Kristie Rebecca Weir, Martina Zangger, Samantha Chan, Flurina Jenal, Marie Roumet, Yvonne Mattmann, Christof Bieri, Carole Elodie Aubert, Nicolas Rodondi, Sofia Carolina Zambrano Ramos, Sven Trelle, Stefan Neuner-Jehle, Pascal Juillerat, Michaela Barbier, Jennifer Inauen, Sven Streit, Katharina Tabea Jungo, Enriqueta Vallejo-Yagüe

**Affiliations:** 1 Institute of Primary Health Care (BIHAM), University of Bern, Bern, Switzerland; 2 Institute of Psychology, University of Bern, Bern, Switzerland; 3 Sydney School of Public Health, Faculty of Medicine and Health, The University of Sydney, Syndey, New South Wales, Australia; 4 Department of Clinical Research, University of Bern, Bern, Switzerland; 5 Institute of Social and Preventive Medicine (ISPM), University of Bern, Bern, Switzerland; 6 Department of General Internal Medicine, Inselspital, Bern University Hospital, Bern, Switzerland; 7 Institute of Primary Care, University of Zurich and University Hospital of Zurich, Zurich, Switzerland; 8 Center for Primary and Community Care, University of Luzern, Luzern, Switzerland; 9 Gastroenterology, Clinic for Visceral Surgery and Medicine, Inselspital, Bern University Hospital, University of Bern, Bern, Switzerland; 10 Crohn and Colitis Center, Gasteroenterology Intesto, Bern and Fribourg, Switzerland; 11 Health Economics Facility, Departement of Public Health, University of Basel, Basel, Switzerland; 12 Institute of Pharmaceutical Medicine (ECPM), University of Basel, Basel, Switzerland; 13 Center for Healthcare Delivery Sciences (C4HDS) and Division of Pharmacoepidemiology and Pharmacoeconomics, Department of Medicine, Brigham and Women's Hospital and Harvard Medical School, Boston, Massachusetts, USA

**Keywords:** Primary Care, Gastroenterology, Medication Review, Multimorbidity, Patient Reported Outcome Measures

## Abstract

**ABSTRACT:**

**Objectives:**

Proton pump inhibitors (PPIs) are widely prescribed medications and commonly used for the treatment of gastric acid-related disorders. Nevertheless, PPIs are often overused leading to potential adverse effects and unnecessary healthcare costs. Deprescribing strategies have emerged to safely reduce or substitute inappropriate PPIs and optimise patient care in an evidence-based manner. This protocol describes a study to evaluate the effectiveness of a PPI deprescribing intervention in comparison to usual care in the Swiss primary care setting.

**Design:**

An open-label, cluster randomised controlled trial.

**Setting:**

Swiss primary care settings.

**Participants:**

Included participants will be adults with inappropriate PPI treatment and will be recruited by general practitioners (GPs). Participants treated by the same GP constitute a cluster. Clusters are randomised 1:1 to either the intervention group or the control group.

**Interventions:**

The intervention components consist of deprescribing tools including educational material, decision aids for both participants and GPs, and additional trainings for GPs only. Patients in the control group will receive usual care. Data will be collected at baseline, 3-, 6-, 9- and 12-month follow-up time through online surveys or a phone call for both GPs and participants.

**Primary and secondary outcome measures:**

The first co-primary endpoint is the effectiveness of the deprescribing intervention measured by the change of prescribed PPI dose. The second co-primary endpoint is safety, which is measured with the Reflux Disease Questionnaire assessing change in gastrointestinal symptoms. There are several secondary endpoints, such as the total number of prescribed medications, occurrences of changes in prescription patterns, PPI discontinuation and cost-effectiveness.

**Conclusions:**

The findings from this study will provide evidence on the effectiveness and safety of a PPI deprescribing intervention for patients and GPs. Successful implementation of our PPI deprescribing strategy has the potential to improve patient outcomes and lower costs.

**Trial registration number:**

NCT06129474.

**STRENGTHS AND LIMITATIONS OF THIS STUDY**

- This trial will test a context-specific, patient-centred intervention that draws on health psychology and behaviour change principles to support general practitioners (GPs) and patients in discontinuing proton pump inhibitors.
- Our intervention is designed to encourage and promote shared decision-making between patients and GPs.
- This trial encourages GPs to be inclusive and seek diversity among their recruited participants.
- We acknowledge that this trial would require less resources and less time from participants, GPs and other team members if there were electronic healthcare records databases available, but these are currently not available in Switzerland.
- Due to the nature of the intervention this trial will be open-label, which could introduce bias.

## Introduction

 Proton pump inhibitors (PPIs) are currently among the most frequently prescribed medications for the management of gastric acid-related disorders.^1^ Among others, they are used to treat gastro-oesophageal reflux disease (GORD), dyspepsia and reflux esophagitis, or for the treatment of ulcers associated with the use of non-steroidal anti-inflammatory medication (NSAIDs), and finally for *Helicobacter pylori* eradication in combination with antibiotics.^2^,^4^ According to a nationwide Swiss study, PPI prescription increased from 19.7% in 2012 to 23.1% in 2017.^4^ One-fourth of prescribed PPIs were characterised as inappropriate, either due to inadequate indication or too high doses.^4^ While PPIs are generally considered to have good short-term tolerance and an excellent safety profile^5^; the long-term use of PPIs is associated with adverse events, such as nutritional deficiencies,^6^,^8^ osteoporosis,^9^ fractures,^10^ intestinal infections with *Clostridioides difficile*, *Shigella*, *Campylobacter* or *Salmonella*,^11 12^ pneumonia,^13^ cancer^14 15^ and increased all-cause readmission,^16^ and healthcare costs.^17 18^

Deprescribing, which involves withdrawing, reducing the dose or substituting medications that are no longer beneficial or potentially harmful, is a well-established strategy for optimising medication use and minimising adverse effects.^19^ Despite the potential benefits of deprescribing PPIs, they are often not deprescribed in routine practice. A case vignette study in 31 countries found that PPIs were among the most common medications that general practitioners (GPs) were willing to deprescribe.^20^ A previous systematic review investigating different deprescribing strategies, reported that implementing a deprescribing intervention led to PPI discontinuation rates ranging between 14% and 64% without deteriorating symptoms.^21^ However, the studies included in this systematic review varied considerably in terms of study design and deprescribing interventions.^21^ Another study evaluating PPI deprescribing strategies in primary care highlighted two key factors associated with the success of the strategies (1) clarity and simplicity of the materials that patients receive and (2) training of the physicians.^22^ In addition, the acceptance of deprescribing depends highly on the setting, country and cultural attitudes towards medication,^23^ which underlines the importance of context-specific strategies including shared decision making between patients and their physicians.

Following the observed increase in inappropriate PPI prescriptions in Switzerland, a context-specific deprescribing intervention is expected to benefit healthcare and reduce unnecessary safety risks in patients.^24^ Therefore, we have developed a context-specific, patient-centred intervention that draws on health psychology and behaviour change principles to support GPs and patients in discontinuing PPIs, which we plan to test in a cluster-randomised clinical trial. The primary objective of our trial is to assess if the study intervention leads to the deprescribing of inappropriate PPIs (ie, effectiveness), while ensuring non-inferiority safety, in comparison to usual care, in adults in the Swiss primary care setting. Second, we aim to investigate the impacts of the intervention on other clinical outcomes, such as the number of medications used, quality of life (QoL), additional safety endpoints and costs. Alongside the trial, a cost-effectiveness analysis will be performed.

## Methods

### Methods: participants, interventions and endpoints

#### Trial design and setting

The DepRescribing inapprOpriate Proton Pump InhibiTors (DROPIT) trial is an open-label, cluster-randomised controlled trial conducted in primary care settings of German-speaking regions in Switzerland.

#### Study participants

Patients eligible for the DROPIT trial will receive regular care from a GP, be ≥18 years old, have sufficient knowledge of German and have a daily PPI intake for ≥8 weeks.

Exclusion criteria include a life-expectancy <12 months (as estimated by the GP), inability to provide informed consent by themselves or a legal representative, taking PPI in an appropriate dose and with an established indication (online supplemental A table S1 and online supplemental A table S2, online supplemental B patient informed consent forms). Similarly, patients are excluded if they have ≥2 of the following medications, or if they are using ≥1 of the following medications and have ≥1 of the risk factors mentioned below. Medications: chronic use of NSAID >7 days, antiplatelet therapy, additional antiplatelet therapy (eg, ticagrelor or similar), anticoagulation, systemic steroids >1 month. Risk factors: history of gastrointestinal ulcer, age ≥65 years, additional use of selective serotonin reuptake inhibitor or serotonin and norepinephrine reuptake inhibitor, severe concomitant disease with increased risk of gastrointestinal bleeding according to the GP’s assessment (ie, severe liver disease, neoplasia, severe misuse of alcohol and nicotine).

#### Study arms

##### Intervention components

**Figure 1:**
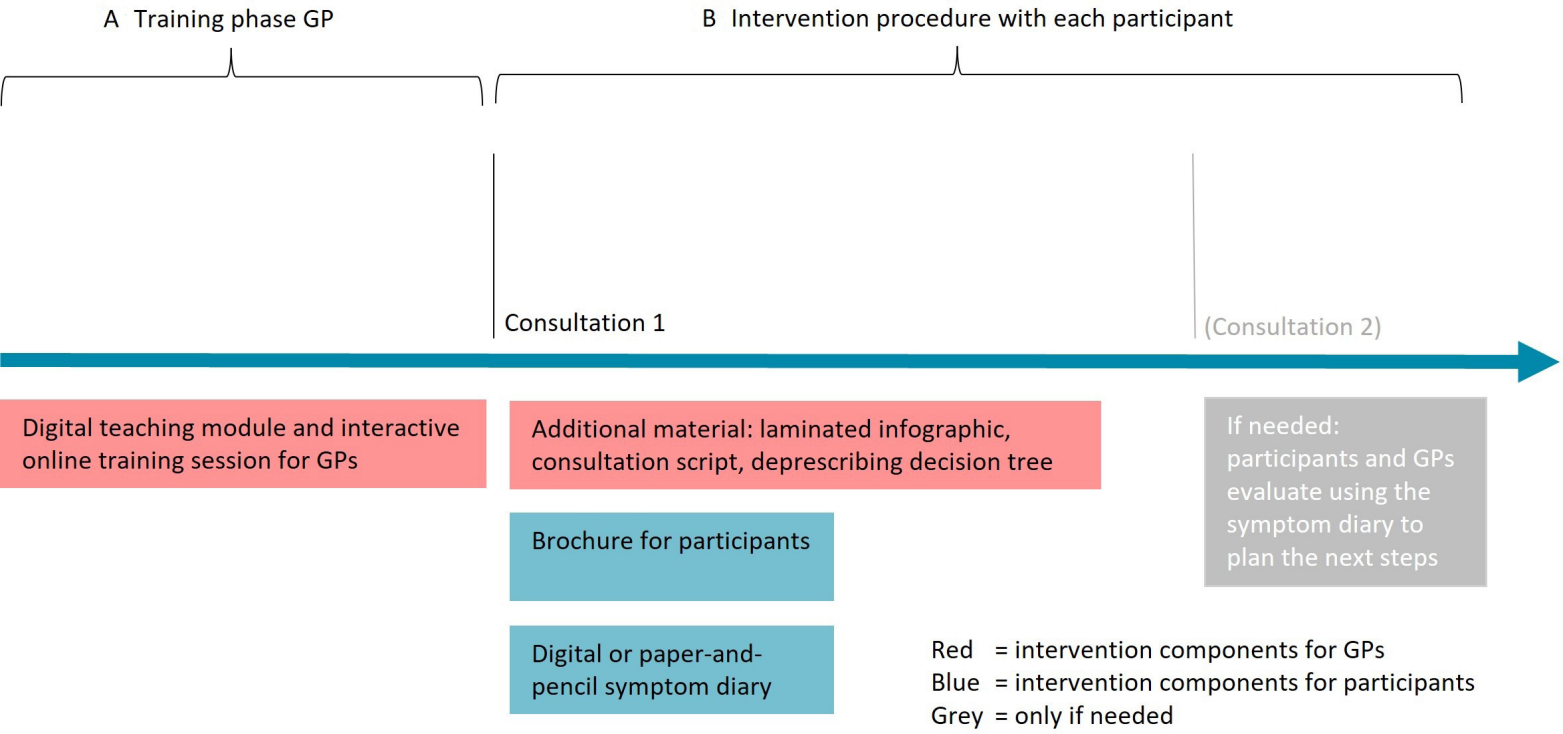
Step-by-step implementation of the intervention.

The GPs in the intervention group receive the PPI deprescribing intervention tools and materials at the start of follow-up. The intervention is designed for the Swiss primary care setting and includes specific materials and tools for both GPs and trial participants (figure 1). It was developed to address the barriers and enablers of PPI deprescribing at patient and GP levels, and the materials were co-designed and adapted based on patient and GP feedback.^25^,^28^ For GPs, the intervention includes an initial digital and printable teaching module that provides comprehensive knowledge and skills related to PPI medications and the deprescribing process. This is followed by an online training session featuring interactive role plays to simulate various GP-patient scenarios. Additional material includes a laminated infographic, a consultation script and a deprescribing decision tree, all designed to assist GPs in guiding participants through the PPI deprescribing process. GPs will also be provided with a brochure to give to the trial participants (patients). This brochure contains information about PPIs, an explanation of the deprescribing process, a list of evidence-based alternative strategies to manage potential rebound symptoms and an invitation to use a digital symptom diary to self-monitor their symptoms and experiences throughout the trial. The digital diary requires internet access and a connection to the calendar on a cell phone or computer. For participants without internet access or a cell phone, a paper-and-pencil version of the symptom diary will be available.

On receipt of the intervention elements, the GPs are expected to use the teaching module, participate in the online training session, evaluate the participant’s PPI prescription and consult with the participant if needed (for which additional materials are provided). An overview of this process is depicted in figure 1.

##### Usual care

Participants and GPs in the control group receive and provide usual care, respectively, and do not have access to the intervention components. As part of standard care, GPs in the control group may evaluate and adjust their patients’ medications at their discretion.

### Follow-up

Follow-up will start the day of the delivery of the intervention (ie, delivery of the intervention materials to GPs), which should be within 30 days after cluster randomisation. In the control group, follow-up will start 15 days after cluster randomisation. Participants in both groups will be followed for 1 year, with data collection occurring at baseline and at 3, 6, 9 and 12 months after the start of follow-up. A detailed timeline for both trial arms is shown in online supplemental A figure S1.

### Study endpoints

#### Co-primary endpoints

There are two co-primary endpoints.

- The first co-primary endpoint (superiority endpoint), evaluating the effectiveness of the intervention, is defined by the prescribed PPI dose as one minus the ratio between the average PPI prescription over the 12-month follow-up and baseline.
- The second co-primary endpoint evaluates safety (non-inferiority) using the Reflux Disease Questionnaire (RDQ). The RDQ is a self-administered questionnaire (12 questions) in which participants are asked to report the frequency and severity of their upper gastrointestinal symptoms using a 6-point scale ranging from 0 (no occurrence) to 5 (daily/severe) points per symptom. The RDQ includes three subscales: regurgitation, heartburn and dyspepsia. For the purpose of this endpoint, the dyspepsia and GORD (regurgitation combined with heartburn) subscales will be used.^29 30^

#### Secondary endpoints

The secondary endpoints are multifaceted: total number of medications prescribed during the trial measured using unique Anatomical Therapeutic Chemical codes, reduction of at least 50% of the prescribed PPI dose, PPI discontinuation (defined as a stop in PPI prescription as indicated by the GP), PPI sustained discontinuation (defined as 3 months without prescribed PPI), a switch to on-demand use, or use of alternative anti-reflux treatments, individual components of the RDQ (regurgitation, heartburn, dyspepsia) and atypical gastrointestinal symptoms measured by the Reflux Symptom Index (RSI).^31^ The RSI scales for each individual item ranges from 0 (no problem) to 5 (severe problem) points, with a maximum of 45 points. Further, we are interested in the occurrences of additional safety outcomes such as ulcers and/or gastrointestinal bleedings, nutritional deficiencies due to PPI overuse, such as vitamin B12, iron, magnesium or sodium, as well as other comorbidities, such as osteoporosis, small intestinal bacterial overgrowth, anaemia, fractures, nephritis and intestinal infections as reported by the GP. Furthermore, we will measure the QoL using the European Quality of Life-5-Dimensions 5-Levels (EQ-5D-5L) questionnaire.^32^ Each of the five dimensions (mobility, self-care, usual activities, pain/discomfort and anxiety/depression) has five levels (no, slight, moderate, severe and extreme problems) out of which one overall utility measure on a scale of 0 (worst health/death) to 1 (perfect health) will be derived using published algorithms.

#### Other endpoints

Additional endpoints will be explored and reported separately from the main trial findings, as detailed in the Dissemination section. We will evaluate cost-effectiveness by relating differences in costs and patients’ QoL over time between the new PPI deprescribing intervention and the usual care control group. Differences in direct medical costs from a Swiss statutory health insurance perspective (main analysis) during the 12-month trial time horizon will be set in relation to the difference in quality-adjusted life-years (QALYs). QALYs will be derived as the area under the survival curve, resulting from utility values calculated from EQ-5D-5L questionnaire responses. Direct medical costs will be based on healthcare resource utilisations as recorded in this trial and publicly available sources of Swiss unit costs. For costs of outpatient specialist visits, cost estimates provided by a Swiss health insurer are available. Medical resources will include but are not limited to GP and specialist consultations, endoscopies, emergency room visits, planned and unplanned hospitalisations, length of hospital stays, rehabilitation stays or any outpatient therapy visits, and/or nursing visits at home. Indirect costs arising from sick leave or reduction of employment due to worsening of symptoms will be considered in a scenario analysis.

### Study timeline

**Figure 2:**
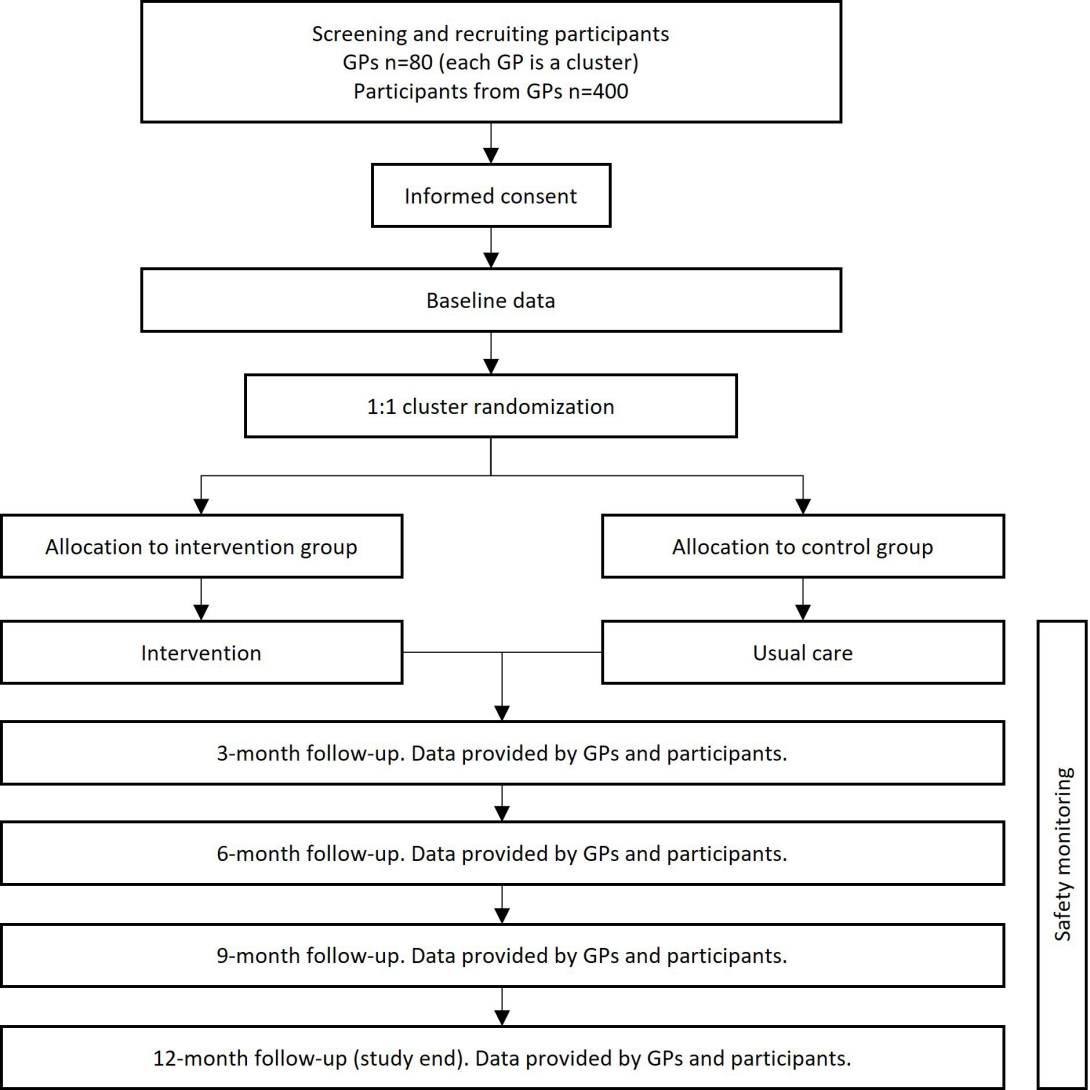
Flowchart of the DepRescribing inapprOpriate Proton Pump InhibiTors (DROPIT) trial from screening to study end.

The duration of the entire project will be 60 months (5 years), with a 28-month recruitment period (ie, time from first patient included, to last patient included) and a follow-up of 12 months per participant. The study timeline is depicted in the flow chart (figure 2).

### Sample size

The sample size calculation was based on a sequential testing procedure in line with the two co-primary endpoints (effectiveness and safety). For the effectiveness, we based the calculations on PPI discontinuation rates from previous literature^33^,^37^ (10% control group, 25% intervention group) and a Pearson χ^2^ test for superiority without continuity correction and the following assumptions: intracluster correlation coefficient of 0.05^38 39^ and a two-sided alpha of 0.05. Finally, we checked whether we achieved adequate power with the estimated sample size to demonstrate a low to medium effect size (Cohen’s d) of 0.4 for the effectiveness endpoint. For the safety, a Student’s t-test was used with the following assumptions: similar values in the two RDQ subscales (dyspepsia and GORD) per patient in both groups at the end of the trial, a non-inferiority margin of 0.5 points,^29^ a common SD of 1.3 resulting in an effect size (Cohen’s d) of 0.38, an intracluster correlation coefficient of 0.06 and a one-sided alpha of 0.025. For both co-primary endpoints, we additionally assumed a total of 80 clusters, a power of 90%, coefficient of variation of the cluster size of 0.5. We calculated a required mean cluster size of 4.0 and 4.5, and a sample size of 316 and 360 patients for the effectiveness and safety co-primary endpoints, respectively. To account for some missing data, we aim to recruit 400 patients who will be recruited in at least 80 clusters with a cluster size of between 2 and 12 participants.

### Recruitment and informed consent

GPs will screen potential participants based on the eligibility criteria during consultations or by reviewing their electronic medical records. They will ask their eligible patients if they would like to be contacted by a member of the study team. Following their acceptance, the study team will give the potential participants a call and guide them through the informed consent process. Subsequently, interested eligible patients will sign and send the informed consent to the study team by post. Once the informed consent is signed by the patient and principal investigator, the patient will be enlisted in the trial.

### Methods: assignment of interventions

#### Cluster-randomisation and group allocation

Each GP is considered as one cluster. Once a cluster is closed (ie, no more recruitment from that GP), GPs and their patients (ie, participants) are randomly assigned in a 1:1 ratio to either the intervention or the control group.

#### Blinding procedures

Given the nature of the intervention, blinding of GPs and patients will not be feasible. Thus, this is an open-label trial. However, those in the control group will remain unaware of the detailed nature of the intervention. Also, the study members involved in data collection, data analysis and statistics will not be blinded to the intervention assigned to each study arm, since information on the implementation of the intervention elements will only be collected from the intervention group.

### Methods: data collection, management and analysis

#### Data collection

Data will be collected from participants and GPs at baseline and at 3-, 6-, 9- and 12-month follow-up. Baseline data will be collected within 60 days prior to group allocation (ie, cluster randomisation). Follow-up data from participants will be collected directly through surveys (operationalised by REDCap^40 41^), or alternatively by phone, based on participants’ preference. Data from GPs will be gathered at the same time points via online surveys (REDCap).

Data collected from patients will include demographics, characteristics, attitudes toward medications, endpoint data, assessment of the implementation of the intervention elements, healthcare utilisation and safety data. From GPs, we will collect eligibility confirmation, information on patients’ medications and diagnoses, assessment of the implementation of the intervention elements and reports of serious adverse events (SAEs).

#### Data management

The data collected will be securely stored in a dedicated electronic clinical data management system (REDCap database^40 41^), with access restricted to authorised personnel only. Participants’ data will be pseudonymised, with personally identifiable information stored separately, except for email addresses used for automatic online survey distribution. In the event of study withdrawal or on study completion, the email addresses used for the automatic survey release will be removed. Regular data reviews will ensure the quality and completeness of the collected data. After the legally required retention period, the study data will be fully anonymised.

#### Statistical analysis

##### Statistical analyses for co-primary endpoints

To address the effectiveness, we will first quantify the change in prescribed PPI dose over the 12-month follow-up period. This will be done in two steps: (1) estimating the average PPI dose over the 12 months of follow-up, by dividing the area under the curve by the observation time and (2) calculating the relative change from baseline as one minus the ratio between the average prescription and the baseline prescription. Subsequently, the difference in the change in prescribed PPI dose between the control and intervention group will be calculated using a linear model, adjusted for the baseline dose and including a random effect for the cluster. We will use an intention-to-treat (ITT) analysis as primary analysis, and a per-protocol analysis as a secondary approach.

To address safety, the difference in the change in upper gastrointestinal symptoms between the two groups will be calculated with a repeated-measures linear mixed-effects model, adjusted for baseline values, and including the intervention group, the timepoint, and the interaction between group and timepoint as fixed effects. Effects of the cluster and participant will be added as random effects. We will conduct both ITT and per-protocol analyses. To claim success for the safety co-primary endpoint, both ITT and per-protocol analyses need to meet non-inferiority.

##### Statistical analyses for secondary and other endpoints

Repeatedly measured continuous secondary endpoints will be analysed using the same model as the safety co-primary endpoint. Count endpoints will be analysed with a generalised linear mixed model and a negative binomial distribution, accounting for the cluster’s random effect and observation time as an offset. Binary endpoints assessed at the end of the follow-up period will be analysed using mixed-effects logistic regression, adjusted for observation time, and including the cluster’s random effect. Cost-effectiveness analysis will employ generalised structural equation models. Finally, a descriptive summary of all pre-specified SAEs and deaths will be provided.

##### Handling of missing data

Even though we assume very few missing data, if there is a substantial amount of missing data, we will consider multiple imputation by chained equation and analysing the available cases as a sensitivity analysis. If the missingness is limited and expected to not confound the interpretation of results, we will disregard the missing data and perform an analysis of the available cases.

### Methods: safety monitoring and public and patient involvement

#### Safety monitoring

Collection, reporting and investigation of SAEs, as well as notification to the ethical committee will be conducted following the applicable regulation and approved process. Conversely to SAEs, reporting of non-SAEs is not mandatory and will be done at the discretion of the treating physician.

To ensure the safety of our participants, we plan to conduct one interim analysis after the first 200 participants are followed for at least 6 months after inclusion. The safety parameters that will be considered in this interim analysis include the RDQ, gastrointestinal ulcers, gastrointestinal bleeding and all SAEs in each group. The advisory board will evaluate unblinded data and provide recommendations.

In addition to the trial’s safety monitoring, we have included relevant adverse events within the primary and secondary safety endpoints.

#### Patient and public involvement

The study is supported by both an advisory board and a stakeholder group. The advisory board is composed of experts in PPI (de)prescribing. They offer guidance on study design, provide perspectives on specific elements, and serve as an independent data safety committee, evaluating participant safety and advising on trial modifications or termination if safety concerns arise. The stakeholder group, made up of clinical professionals and patient representatives, provides input on the trial design and intervention materials aimed at GPs and participants. Additionally, the DROPIT intervention has been developed with repeated input from patients/citizens and GPs.

## Discussion

The findings from this study will provide evidence on the effectiveness and safety of the DROPIT PPI deprescribing intervention. Successful implementation of our PPI deprescribing strategy could improve patient outcomes by reducing unnecessary adverse effects, such as infections, fractures or nutrient deficiencies, while ensuring appropriate, evidence-based treatment. Additionally, it could promote rational medication use, reduce polypharmacy, and minimise healthcare costs and resource utilisation. To our knowledge, this is the first randomised controlled PPI deprescribing trial in the primary care setting of Switzerland.

We chose a cluster-study design to prevent contamination that could arise if GPs were to treat participants in both the intervention and control groups.^42^ However, we recognise that cluster trials can introduce selection bias, as participant recruitment typically occurs after randomisation.^43 44^ To mitigate this, recruitment in our trial will cease once a cluster is closed, after which participants will be assigned to the intervention or control group based on their GP’s random assignment. It is unlikely that patients will avoid visiting their GP due to prior knowledge of the trial.

The 12-month follow-up period is consistent with similar studies and should be sufficient to capture potential rebound symptoms.^45^,^48^ Nevertheless, there is a small risk that we might miss the reoccurrence of GI bleeding or ulceration that might occur at a later time point, mainly because we are following the participants from the time we deliver the intervention to the GPs, which does not necessarily coincide with the timing of deprescribing. However, with a 12-month follow-up, and expecting that deprescribing will be done earlier rather than later during that time, we expect to observe the participant after the deprescribing.

Finally, we recognise that participants may receive new diagnoses during the follow-up period that justify PPI use, potentially no longer fulfilling the initial eligibility criteria. However, since this reflects typical clinical variations and occurs after randomisation, we will not exclude these participants. Instead, we may conduct a sensitivity analysis for the co-primary endpoints, where these participants will be censored.

### Strengths

The deprescribing strategy targets both GPs and patients, which has shown in other deprescribing strategies to be more effective than being solely designed for GPs.^49^ Therefore, deprescribing in the trial will involve shared decision-making between participants and GPs’ and thus aids to empower patients to participate actively in their treatment and promote their autonomy.

Population wise, we aim for diversity and inclusion in our trial. Thus, we encourage GPs to be inclusive and seek diversity among their recruited participants (eg, sex, age, ethnicity). Additionally, since PPIs can be used in pregnant and breastfeeding women, we will not exclude this group.

Finally, this study aims to benefit from well-conducted scientific research and is supported by a multidisciplinary team, along with an advisory board and stakeholder group.

### Limitations

Neither participants nor GPs in the intervention group or study team members/data analysts will be blinded due to the trial design. The data collected through the online survey by the participants heavily depend on the response rate. To the best of our ability, we will try to send online reminders to both participants and GPs to complete the survey.

One could hypothesise that the GPs participating in a deprescribing study may be more attentive to reducing inappropriate prescribing. However, in the study by Vidonscky Lüthold *et al* from our team, we observed that when GPs were instructed to actively identify inappropriate PPIs in patient records, only 35% of the inappropriate PPIs were stopped or reduced after 12 months, while over 60% remained unchanged.^24^ Reasons for a failed deprescribing included lack of discussion with patients, their unwillingness to stop, and fear of recurrence of symptoms. Therefore, it cannot be excluded that an increased awareness among control group patients will bias the trial results towards the null, but the effect is likely to be small.

Each GP is treated as a separate cluster and GPs are requested to keep confidentiality with colleagues. This is particularly relevant when several trial GPs work in the same practice. Otherwise, a contamination across GP clusters may potentially bias the trial results toward the null hypothesis, underestimating the intervention effect. However, GPs are used to maintaining confidentiality among colleagues and have little time to discuss the trial with peers, thus we trust that group contamination will be minimal.

In Switzerland, low dose PPI are available over the counter in pharmacies. While we will ask participants to report any self-administered medications that were not prescribed, we cannot completely control whether they purchase and use PPIs independently without reporting them. However, this issue is not expected to differ between the two groups.

There might be the possibility of a slight reporting bias as the participant surveys include questions about upper gastrointestinal symptoms. Therefore, participants could be more aware of upper gastrointestinal problems during the trial period and report these more frequently.

Furthermore, GPs are encouraged to join the trial through various channels such as quality circles (ie, educational meetings for GPs), distributing flyers and emails via GP networks or personal contacts. Although the network is widely spread within the German-speaking part of Switzerland, it does not fully represent the entire GP population since the French- and Italian-speaking parts are not covered.

Finally, we acknowledge that this trial would require less resources and less time from participants, GPs, and other team members if there were electronic healthcare records databases available, instead of having to collect all information from scratch, de novo. Unfortunately, this is not available in the Swiss healthcare system.

## Conclusion

We expect that our deprescribing strategy will effectively reduce inappropriate PPIs, but sustained benefits after the trial will only be possible if the community embraces the intervention in usual practice. Our trial is expected to demonstrate the effectiveness and safety of this multi-faceted intervention, which is contextualised for GPs and patients to deprescribe PPIs. If successful, we aim to widely distribute the intervention materials and tools across Switzerland, as we have designed the intervention with the real-world setting in mind. This could be a valuable tool to reduce unnecessary adverse effects and minimise healthcare costs on a national level.

### Current status of the DROPIT trial

First participant is planned for Autumn 2024.

## Ethics

We greatly value and respect the contributions of those who share their clinical data and insights from practice with us. We are fully committed to upholding their rights, which include ensuring privacy, obtaining informed consent (online supplemental B), and respecting their right to withdraw from the study at any time. GPs who recruit patients are financially compensated for their time and expertise as healthcare professionals involved in the study. While many patients join the study out of a desire to contribute to research, we also expect that, as users of PPI medication, they may experience direct benefits from participating. The risks in this study are minimal, as deprescribing is part of routine clinical practice, and the study intervention does not require any measures beyond this. Resuming medication is possible at any time during the study, and participants can withdraw from the study at any time. Additionally, in our efforts to create social value, we actively involve a range of stakeholders in our decision-making processes to ensure that diverse perspectives are considered (see the Patient and public involvement section). The study will be conducted in accordance with principles in the current version of the Declaration of Helsinki,^50^ the relevant parts of the guidelines of Good Clinical Practice (GCP) from the International Council for Harmonisation (ICH), as well as local regulations. This study has been approved by the Ethics Committee of Bern, Switzerland (BASEC ID: 2024-00685).

### Dissemination

The DROPIT trial follows an open access policy and aims for comprehensive dissemination of all resulting data through several publications, including primary and secondary outcomes, and cost-effectiveness analysis and intervention development. Additionally, we will publish other articles on the qualitative evaluation of the intervention’s acceptability, which is an embedded study and not described in this protocol manuscript. In addition to publishing in international journals, we plan to share findings with the broader public, including lay press, and present at public conferences and symposia involving GPs and hospital physicians.

### Administration

We wrote this study protocol in concordance with the Standard Protocol Items: Recommendations for Interventional Trials.^51 52^

## supplementary material

10.1136/bmjopen-2024-094495 online supplemental file 1

10.1136/bmjopen-2024-094495 online supplemental file 2

## References

[1] Shanika LGT, Reynolds A, Pattison S (2023). Proton pump inhibitor use: systematic review of global trends and practices. Eur J Clin Pharmacol.

[2] Katz PO, Dunbar KB, Schnoll-Sussman FH (2022). ACG Clinical Guideline for the Diagnosis and Management of Gastroesophageal Reflux Disease. Am J Gastroenterol.

[3] Luo X, Hou M, He S (2022). Efficacy and safety of concomitant use of proton pump inhibitors with aspirin-clopidogrel dual antiplatelet therapy in coronary heart disease: A systematic review and meta-analysis. Front Pharmacol.

[4] Muheim L, Signorell A, Markun S (2021). Potentially inappropriate proton-pump inhibitor prescription in the general population: a claims-based retrospective time trend analysis. Therap Adv Gastroenterol.

[5] Strand DS, Kim D, Peura DA (2017). 25 Years of Proton Pump Inhibitors: A Comprehensive Review. Gut Liver.

[6] Choudhury A, Jena A, Jearth V (2023). Vitamin B12 deficiency and use of proton pump inhibitors: a systematic review and meta-analysis. Expert Rev Gastroenterol Hepatol.

[7] Lam JR, Schneider JL, Zhao W (2013). Proton pump inhibitor and histamine 2 receptor antagonist use and vitamin B12 deficiency. JAMA.

[8] Srinutta T, Chewcharat A, Takkavatakarn K (2019). Proton pump inhibitors and hypomagnesemia: A meta-analysis of observational studies. Medicine (Balt).

[9] Ito T, Jensen RT (2010). Association of long-term proton pump inhibitor therapy with bone fractures and effects on absorption of calcium, vitamin B12, iron, and magnesium. Curr Gastroenterol Rep.

[10] Poly TN, Islam MM, Yang H-C (2019). Proton pump inhibitors and risk of hip fracture: a meta-analysis of observational studies. Osteoporos Int.

[11] Deshpande A, Pant C, Pasupuleti V (2012). Association between proton pump inhibitor therapy and Clostridium difficile infection in a meta-analysis. Clin Gastroenterol Hepatol.

[12] Haastrup PF, Thompson W, Søndergaard J (2018). Side Effects of Long-Term Proton Pump Inhibitor Use: A Review. Basic Clin Pharmacol Toxicol.

[13] Nguyen PA, Islam M, Galvin CJ (2020). Meta-analysis of proton pump inhibitors induced risk of community-acquired pneumonia. Int J Qual Health Care.

[14] Koyyada A (2021). Long-term use of proton pump inhibitors as a risk factor for various adverse manifestations. Ther.

[15] Sasaki T, Mori S, Kishi S (2020). Effect of Proton Pump Inhibitors on Colorectal Cancer. Int J Mol Sci.

[16] Aubert CE, Blum MR, Gastens V (2023). Prescribing, deprescribing and potential adverse effects of proton pump inhibitors in older patients with multimorbidity: an observational study. CMAJ Open.

[17] Ladd AM, Panagopoulos G, Cohen J (2014). Potential costs of inappropriate use of proton pump inhibitors. Am J Med Sci.

[18] Mucherino S, Casula M, Galimberti F (2022). The Effectiveness of Interventions to Evaluate and Reduce Healthcare Costs of Potentially Inappropriate Prescriptions among the Older Adults: A Systematic Review. Int J Environ Res Public Health.

[19] Reeve E, Gnjidic D, Long J (2015). A systematic review of the emerging deﬁnition of “deprescribing” with network analysis: implications for future research and clinical practice. Br J Clin Pharmacol.

[20] Jungo KT, Mantelli S, Rozsnyai Z (2021). General practitioners’ deprescribing decisions in older adults with polypharmacy: a case vignette study in 31 countries. BMC Geriatr.

[21] Haastrup P, Paulsen MS, Begtrup LM (2014). Strategies for discontinuation of proton pump inhibitors: a systematic review. Fam Pract.

[22] Del-Pino M, Sanz EJ (2023). Analysis of deprescription strategies of proton pump inhibitors in primary care: a narrative review. Prim Health Care Res Dev.

[23] Reeve E, Shakib S, Hendrix I (2014). Review of deprescribing processes and development of an evidence-based, patient-centred deprescribing process. Br J Clin Pharmacol.

[24] Vidonscky Lüthold R, Henz NC, Fuhrer C (2023). Inappropriate proton-pump inhibitor prescribing in primary care - an observational study with quality circles. Swiss Med Wkly.

[25] Raghunath AS, Hungin APS, Cornford CS (2005). Use of proton pump inhibitors: an exploration of the attitudes, knowledge and perceptions of general practitioners. Digestion.

[26] Wermeling M, Himmel W, Behrens G (2014). Why do GPs continue inappropriate hospital prescriptions of proton pump inhibitors? A qualitative study. Eur J Gen Pract.

[27] Boath EH, Blenkinsopp A (1997). The rise and rise of proton pump inhibitor drugs: Patients’ perspectives. Soc Sci Med.

[28] Grime J, Pollock K, Blenkinsopp A (2001). Proton pump inhibitors: perspectives of patients and their GPs. Br J Gen Pract J R Coll Gen Pract.

[29] Shaw M, Dent J, Beebe T (2008). The Reflux Disease Questionnaire: a measure for assessment of treatment response in clinical trials. Health Qual Life Outcomes.

[30] Shaw MJ, Talley NJ, Beebe TJ (2001). Initial validation of a diagnostic questionnaire for gastroesophageal reflux disease. Am J Gastroenterol.

[31] Belafsky PC, Postma GN, Koufman JA (2002). Validity and reliability of the reflux symptom index (RSI). J Voice.

[32] Janssen MF, Pickard AS, Golicki D (2013). Measurement properties of the EQ-5D-5L compared to the EQ-5D-3L across eight patient groups: a multi-country study. Qual Life Res.

[33] Research Ethics Committee (2015). Persons with limited life expectancy. https://www.forskningsetikk.no/en/resources/the-research-ethics-library/research-on-particular-groups/persons-with-limited-life-expectancy.

[34] Wallace LS, Rogers ES, Roskos SE (2006). Brief report: screening items to identify patients with limited health literacy skills. J Gen Intern Med.

[35] Horne R, Weinman J, Hankins M (1999). The beliefs about medicines questionnaire: The development and evaluation of a new method for assessing the cognitive representation of medication. Psychol Health.

[36] World Health Organization (2021). Defined daily dose (DDD): definition and general considerations.

[37] Craig P, Dieppe P, Macintyre S (2008). Developing and evaluating complex interventions: the new Medical Research Council guidance. BMJ.

[38] Moore GF, Audrey S, Barker M (2015). Process evaluation of complex interventions: Medical Research Council guidance. BMJ.

[39] Zhang C-Q, Zhang R, Schwarzer R (2019). A meta-analysis of the health action process approach. Health Psychol.

[40] Harris PA, Taylor R, Minor BL (2019). The REDCap consortium: Building an international community of software platform partners. J Biomed Inform.

[41] Harris PA, Taylor R, Thielke R (2009). Research electronic data capture (REDCap)--a metadata-driven methodology and workflow process for providing translational research informatics support. J Biomed Inform.

[42] Magill N, Knight R, McCrone P (2019). A scoping review of the problems and solutions associated with contamination in trials of complex interventions in mental health. BMC Med Res Methodol.

[43] Hahn S, Puffer S, Torgerson DJ (2005). Methodological bias in cluster randomised trials. BMC Med Res Methodol.

[44] Puffer S, Torgerson D, Watson J (2003). Evidence for risk of bias in cluster randomised trials: review of recent trials published in three general medical journals. BMJ.

[45] Nguyen-Soenen J, Rat C, Gaultier A (2022). Effectiveness of a multi-faceted intervention to deprescribe proton pump inhibitors in primary care: protocol for a population-based, pragmatic, cluster-randomized controlled trial. BMC Health Serv Res.

[46] Clyne B, Smith SM, Hughes CM (2015). Effectiveness of a Multifaceted Intervention for Potentially Inappropriate Prescribing in Older Patients in Primary Care: A Cluster-Randomized Controlled Trial (OPTI-SCRIPT Study). Ann Fam Med.

[47] Zwisler JE, Jarbøl DE, Lassen AT (2015). Placebo-Controlled Discontinuation of Long-Term Acid-Suppressant Therapy: A Randomised Trial in General Practice. Int J Family Med.

[48] Wehmeyer MH, Horvatits T, Buchholz A (2022). Stop of proton-pump inhibitor treatment in patients with liver cirrhosis (STOPPIT): study protocol for a prospective, multicentre, controlled, randomized, double-blind trial. Trials.

[49] Wilsdon TD, Hendrix I, Thynne TRJ (2017). Effectiveness of Interventions to Deprescribe Inappropriate Proton Pump Inhibitors in Older Adults. Drugs Aging.

[50] World Medical Association (2013). World Medical Association Declaration of Helsinki: ethical principles for medical research involving human subjects. JAMA.

[51] Chan A-W, Tetzlaff JM, Altman DG (2013). SPIRIT 2013 statement: defining standard protocol items for clinical trials. Ann Intern Med.

[52] Chan A-W, Tetzlaff JM, Gøtzsche PC (2013). SPIRIT 2013 explanation and elaboration: guidance for protocols of clinical trials. BMJ.

