# Supplementary material for: DepRescribing inapprOpriate Proton Pump InhibiTors (DROPIT): study protocol of a cluster-randomised controlled trial in Swiss primary care
Source: BMJ Open. 2025 Jan 20;15(1):e094495. doi: 10.1136/bmjopen-2024-094495 (PMC11749314; doi:10.1136/bmjopen-2024-094495)
Supplement: online supplemental file 1 [file bmjopen-15-1-s001.docx]

**SUPPLEMENTARY A**

**Table S1:** Appropriate proton pump inhibitor doses used with an established indication (see exclusion criteria).

| **Substance** | **Dose** |
| --- | --- |
| Pantoprazole | ≤40 mg/d |
| Omeprazole | ≤40 mg/d |
| Lansoprazole | ≤30 mg/d |
| Dexlansoprazole | ≤30 mg/d |
| Esomeprazole | ≤20 mg/d |
| Rabeprazole | ≤20 mg/d |

**Table S2:** Established indications for long-term PPIs (see exclusion criteria).

| - History of bleeding ulcer - Peptic ulcer due to cause other than NSAID or *H. pylori* - Barrett’s oesophagus - Severe erosive reflux disease (Los Angeles grade C/D) - GERD with symptoms or complications (oesophageal ulcer, peptic stricture) - Other indications such as Zollinger-Ellison-Syndrome, PPI-sensitive eosinophilic esophagitis, chronic pancreatitis with steatorrhea refractory to enzyme replacement therapy, idiopathic pulmonary fibrosis |
| --- |


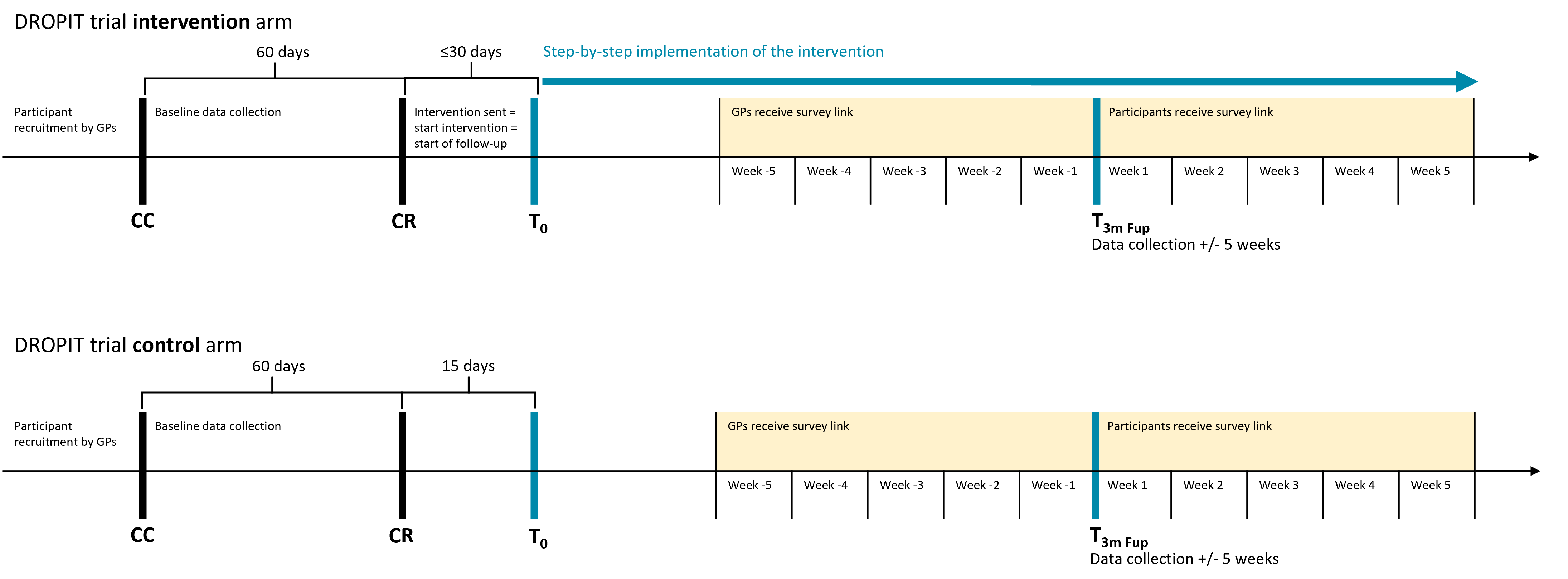


**Figure S1:** Study timeline for each trial arm. Abbreviations used: CC = cluster closing, CR = cluster randomisation, T = Timepoint, 3m FUP = 3-month Follow-up.
