# Supplementary material for: DepRescribing inapprOpriate Proton Pump InhibiTors (DROPIT): study protocol of a cluster-randomised controlled trial in Swiss primary care
Source: BMJ Open. 2025 Jan 20;15(1):e094495. doi: 10.1136/bmjopen-2024-094495 (PMC11749314; doi:10.1136/bmjopen-2024-094495)
Supplement: online supplemental file 2 [file bmjopen-15-1-s002.docx]

**SUPPLEMENTARY B**

- Patient informed consent (PIC) for participants* (v2.0), pages 2-17
- Patient informed consent (PIC) for legal representatives of patients* (v2.0), pages 18-36

*These forms have been translated to English using DeepL for the purpose of this manuscript. The original forms are in German and can be received upon request.

**The DROPIT study:**

**A national clinical study on medication optimisation**

Participant information

Dear Sir or Madam,

Thank you very much for your interest in this study.

We would like to inform you about the DROPIT study and invite you to take part. In this study, we want to analyse your drug therapy.

Your participation is voluntary. The following **information for participants** is intended to help you make your decision. If you are interested in participating, please inform your family doctor. You will then be contacted by telephone at a later date by a representative of the study team to discuss the study with you in detail. Please keep this participant information that you have received from your GP until this telephone call.

**If anything is unclear to you, please ask your general practitioner or your contact person from the study team.** If you would like to continue participating in the study after your discussion with your contact person from the study team, please **sign the consent form** at the end of this information letter. By signing, you confirm that you have read and understood this information for participants**.**

The participant information and declaration of consent consist of four parts:

Part 1 The most important facts in brief
Part 2 This is what it's all about in detail: Information on the study
Part 3 Data protection and insurance cover
Part 4 Declaration of consent

This study is organised by the Bern Institute of Family Medicine (BIHAM) at the University of Bern. This institution is called the sponsor. The sponsor is responsible for the initiation, management and financing of the study. The person responsible on the sponsor's side is Prof. Dr Sven Streit, BIHAM, University of Bern.

**In the context of this study is responsible for you:**

Name Prof Dr med Sven Streit

Address Bern Institute of Family Medicine (BIHAM),
Mittelstrasse 43, 3012 Bern

Telephone +41 31 631 58 70

e-mail [dropit.biham@unibe.ch](mailto:dropit.biham@unibe.ch)

Part 1: The most important facts in brief

Especially from phase 3 studies (IMP) or confirmatory MD studies.

# Why are we conducting this study?

Many people take medication on a regular basis. It may be that certain medications are unsuitable for treatment or that their benefits are insufficient. Taking these medications often has a negative impact on the patient's state of health and quality of life. For this reason, optimising (discontinuing, reducing or restarting) the medication often makes sense. In this study, we are investigating how medication optimisation can work better. You can find out more about the scientific background to the study in Part 2, **Chapter 4.**

# What do you have to do if you take part?

Participation in this study will take you 12 to 15 months. At the beginning, you will be randomly allocated to one of two groups. During this time, we will invite you to complete a questionnaire 5 times (every 3 months). The questionnaire can either be completed electronically by yourself or during a telephone call with someone from the study team. Completing the questionnaire will take about 30 to 45 minutes.

# What are the benefits and risks associated with participation?

## Benefit

The results from this study will help us to improve guidelines for drug optimisation. Direct benefits for you as a study participant cannot be guaranteed.

## Risk

Any changes to the medication are made with the health interests of the participants in mind. Nevertheless, adverse effects can occur. For example, symptoms of an illness may reappear when a medication is discontinued or the dose is reduced. Or new symptoms may occur when taking a new medication or after increasing the dose of a medication currently in use. We are constantly working to minimise these risks as much as possible. In Part 2, **Chapter 6**, you will find further information on risks, side effects and side effects.

Part 2: This is what it's all about in detail: Information on the study

# The scientific background to the study

## Background: Why are we conducting this study?

In the case of drug treatment, it may be that (further) treatment with a medication is not (or no longer) medically appropriate. On the other hand, it can happen for various reasons that patients do not receive medication with a presumed greater benefit. Both scenarios can have negative consequences. It is therefore important to constantly optimise and update the use of medication for patients. The aim of this study is to test / develop a new strategy to support patients and GPs in optimising medication safely and effectively.

## Structure of the study: How do we proceed?

This clinical trial will be conducted in Switzerland from 2023 to 2028. A total of 80 GPs are taking part. This includes your family doctor. Each GP will include around 5 participants, with a total of 400 people taking part in the study.

In the study, the participating GPs are randomly divided into two groups. This is referred to as "randomisation":

- **Group 1 (trial group):** GPs in this group receive instructions on how to optimise the medication list.
- **Group 2 (control group):** GPs in this group receive no guidance and the participants are treated as usual.

Depending on your GP's group assignment, your medication list will either be optimised according to the instructions (**group 1**) or your GP will look after you according to the standard procedure (**group 2**), as would also be the case outside the study.

In this study, you as a participant do not know which group you and your GP have been assigned to. The idea behind this is that you as a participant can consciously or unconsciously have as little influence as possible on the results, which could falsify the study results. The random group assignment of the GPs and the blinding of the participants should help us to be able to assess as objectively as possible at the end of the study whether or how well the optimisation of the medication actually works in this study.

## Regulations on scientific research involving human subjects

We conduct this study in accordance with the laws in Switzerland (Human Research Act, data protection laws). We also observe all internationally recognised guidelines. The responsible ethics committee has reviewed and approved the study.

# Procedure of the study

## What do you have to do if you take part in the study?

Participation in this study is voluntary and lasts between 12 and 15 months. You must adhere to the study schedule and all instructions given to you by the study team and/or your family doctor.

- You should inform your family doctor if your state of health changes, for example if you feel worse or if you have new complaints. This also applies if you discontinue the study prematurely (see chapters 5.3 and 5.4). To ensure the safety of all participants, we would like to track all incidents that affect your health. This is independent of whether or not they are attributable to the study. We therefore ask that you contact your family doctor if you have any health concerns or events.
- As a study participant, it is necessary that you adhere to the requirements of the study. This includes 1) attending all appointments with your family doctor during the course of the study (if possible) for joint consultations (at the request of the family doctor) and 2) answering the study team's questions about your medication intake, your state of health and your quality of life (every 3 months).

## What happens during the appointments?

During the study, your GP may invite you for a visit at any time to discuss your medication.

**Overview of the study program:**

|  | **What happens?** | **Timing** | **Location** | **Time required** |
| --- | --- | --- | --- | --- |
| **1** | Enquiry by GP, if you are interested you will receive the participant information and your GP will pass on your contact details to the study team | today | GP practice | 20 minutes |
| **2** | Telephone call by the study team and explanation of the details of participation, signing of the consent form at the end of this document (study inclusion) and recording of an emergency contact. | approx. 1-2 weeks before study inclusion | at home | 30-45 minutes |
| **3** | Collection of baseline data, this means filling out a questionnaire (online or with the study team). | - | at home | 30-45 minutes |
| **4** | Possible discussion with family doctor  With consent, this meeting can be recorded. | approx. 4 weeks after study inclusion | GP practice | 30-45 minutes |
| **5** | Possible completion of a symptom diary | approx. 2 weeks after the possible consultation with the family doctor | at home | 1-3 minutes per day |
| **6** | Completion of a questionnaire (online or by telephone with the study team) about your state of health, quality of life and medication. | 3 months after study inclusion | at home | 30-45 minutes |
| **7** | Completion of a questionnaire (online or by telephone with the study team) | 6 months after study inclusion | at home | 30-45 minutes |
| **8** | Completion of a questionnaire (online or by telephone with the study team) | 9 months after study inclusion | at home | 30-45 minutes |
| **9** | Completion of a questionnaire (online or by telephone with the study team) | 12 months after study inclusion | at home | 30-45 minutes |
| **Total** | | | | **~4-7 hours** |

## When does participation in the study end?

In total, regular study participation will take you **between 12 and 15 months**.

You can also cancel your participation earlier at any time. You do not have to explain why you no longer wish to participate. If you would like to end your participation earlier yourself, please speak to your GP. Even if you end your participation prematurely, your GP will continue to provide you with medical care in accordance with current standards.

We may also have to ask you to end the study prematurely. This is the case, for example, if your family doctor or the study director comes to the conclusion that your continued participation is no longer in your best (health) interest. It is also possible that the entire clinical trial will be terminated prematurely, meaning that participation ends prematurely for all participants.

If you drop out of the study prematurely, we will contact you one last time by telephone. The data collected up to the point of discontinuation will still be analysed for the study.

## What happens if you don't want to take part?

Even if you do not take part in this study, your medical treatment will be guaranteed regardless of your decision, and your existing treatment will continue unchanged.

## Pregnancy

You may have children during and immediately after the end of the study. This applies to both women and men. You will discuss these questions with your investigator.

# Risks, burdens and side effects

As with any medical treatment, there are risks and burdens associated with participating in this study. Some risks are already known, others are still unknown. This uncertainty is not unusual in a trial environment. For example, symptoms of a disease may reappear when a drug is discontinued or the dose is reduced. Or new symptoms may occur when taking a new drug or after increasing the dose of a drug currently in use. We are constantly working to minimise these risks and will keep you informed of any new findings on risks and side effects during the study. **Overall, the risks do not differ from the usual treatment. All decisions regarding your treatment will be made together with your family doctor.**

# Financing and compensation

This study is organised by the Bern Institute of Family Medicine (BIHAM) and its partners (Institute of Family Medicine Zurich, Institute of Psychology at the University of Bern, Institute of Social and Preventive Medicine Bern, Patient Service Center Basel and Zurich, Swiss Patient Safety Foundation). The study is financed by the Swiss National Science Foundation as part of the "Investigator Initiated Clinical Trials" program.

The researchers involved have no direct financial benefit from conducting this study.

Your appointments with your GP will be billed in the usual way via your health insurance company. For an appointment with your family doctor that is solely about the study, the family doctors will receive compensation from the sponsor and you or your health insurance company will not be charged anything. There are no additional costs for you or your health insurance company for participating in the study.

You will not be financially compensated for participating in the study.

# Results from the study

The overall results of the study, which come from the data of all participants, do not affect you and your health directly. If you provide the study team with your e-mail address, we will be happy to send you a summary of the overall results of the study at the end of the study.

Part 3: Data protection and insurance cover

# Data protection

We protect your data. There are strict legal regulations on data protection in Switzerland.

The Swiss Data Protection Act gives you the right to information, correction and receipt of your data that is collected, processed and forwarded as part of the study. These rights cannot always be guaranteed in exceptional cases due to other legal or regulatory requirements. If you have any questions, please contact your investigator.

## Encryption of data

Each study generates data from the investigations. This data is documented. This is usually done electronically in large tables, the so-called "data collection sheets". In this study, these are primarily data on your medication, your state of health and your quality of life. All data is documented in encrypted form. "Encrypted" means that personal information that can directly identify you is stored separately from the study results. For this purpose, there is a list (key list) that identifies each person with a unique code. Access to the key to this code is restricted and only accessible to authorised study team members. Your general practitioner will be aware of your code, but will not have access to your data in the database. The key list will remain with us at the institution for a period of 10 years and will be kept under the direct responsibility of the study director, after which it will be destroyed. No one else will receive this key list. Special exceptions are regulated in chapter 9.5.

Your personal data that we need to communicate with you (e.g. by post and telephone) will be stored separately from the data in the study database. This data will be deleted immediately after the end of the study. Only your e-mail address will be stored in the database for sending the questionnaires. As soon as the study is completed or you wish to withdraw from the study, the e-mail address will be deleted and will no longer be included in the database.

At the end of the legally prescribed 10-year retention period, the study data will be completely anonymised. This means that it will no longer be possible to link the data to your person without disproportionate effort. Among other things, the key list will be destroyed for anonymisation.

## Secure handling of data during the study

The sponsor is responsible for the safe handling of your data from the study and is responsible for ensuring that the applicable laws, e.g. data protection laws, are complied with. It is often important that your family doctor shares your medical history with the study team. You authorise this by giving your consent at the end of the document.

## Secure handling of data after the study

The sponsor remains responsible for the secure handling of your data even after the end of the study. The law stipulates that all study documents, including the data in the study database, must be kept for at least 10 years.

At the end of this long period, study data remains encrypted. Or: measures are taken to prevent the data from being assigned to you personally as far as possible. Health-relevant data from your medical history, including from this study, are and will always remain accessible to those treating you. Once a study has been completed, the results are usually published in scientific journals. The results are also scrutinised by other experts. Your coded data must be passed on to these specialists. However, the data may not be used for new research purposes. This would require your separate consent (🡪 Chapter 9.4).

## Further use and disclosure of your data in other, future studies

Your data from this study is also very important for future research projects. Data that has already been used, as well as data that has not yet been used for this study, can possibly be used pseudonymised or anonymised for other research projects and / or passed on (also abroad).

We require your separate consent for the further use and / or forwarding of your data. This is voluntary. Please read the additional declaration of consent at the end of the document carefully. Please sign the consent form if you wish to support further research projects with your data in the future. Even if you do not consent to the further use and / or disclosure, you can still participate in the study.

## Inspection rights during inspections

The conduct of this study can be reviewed. This is done by authorities such as the responsible ethics committee. The sponsor must also carry out such reviews to ensure the quality of the study and the results.

A small number of specially trained people are given access to your personal data and medical history for this purpose. The data is therefore not encrypted for this review. The people who see your unencrypted data are subject to a duty of confidentiality.

As a study participant, you have the right to view your data at any time.

# Insurance cover

You are insured if you suffer a loss as a result of participating in the study. The procedure is regulated by law. The sponsor has taken out insurance with Baloise for this purpose. If you believe that you have suffered a loss as a result of the study, please contact your family doctor or the insurance company directly.

Baloise Insurance Ltd
Aeschengraben 21
P.O. Box 2275
CH-4002 Basel

Part 4: Declaration of consent

Please read this form carefully. Please ask us if anything seems unclear or if there is anything else you would like to know.

This consent consists of three independent declarations of consent:

1. Consent to participate in the DROPIT study
2. Consent for the further use of data from this study in encrypted form, e.g. for new research purposes.
3. Consent to record a possible meeting for quality assurance.

### Declaration of consent for participation in the DROPIT study.

| **BASEC number** | 2024-00685 |
| --- | --- |
| **Title of the study:** | The DROPIT study: A national clinical study on medication optimisation |
| **Responsible institution:**  (Sponsor with address) | Prof Dr med Sven Streit  Bern Institute of Family Medicine (BIHAM)  Mittelstrasse 43, 3012 Bern  Tel: +41 31 631 58 70 |
| **Surname and first name GP**  (Indicate in block capitals) |  |
| **Surname and first name of study participant:**  (Indicate in block capitals) |  |
| **Date of birth Study participant:** |  |
| **Gender Study participants:** | female  male  diverse |

- I received verbal and written information about the study from the study team.
- The study team explained the purpose, procedure and risks of the study to me.
- I am taking part in the study voluntarily.
- The study team explained to me what standard treatments are available outside of the study.
- I have had sufficient time to make this decision. I will keep the written information and receive a copy of my written declaration of consent.
- I can cancel my participation at any time. I do not have to explain why. Even if I end my participation, I will continue to receive my medical treatment. The data collected up to that point will still be analysed as part of the study.
- If I withdraw, the data will remain encrypted or anonymised after 10 years. My email address for the automatic sending of questionnaires will be deleted from the database and no further data will be collected from me.
- If it is better for my health, the family doctor or the principal investigator can exclude me from the study at any time.
- My family doctor must know that I am taking part in the study. My GP is authorised to share data from my medical history that is important for the study with the investigator.
- The responsible specialists of the sponsor and the ethics committee may view my unencrypted data for monitoring purposes. All these persons are subject to a duty of confidentiality.
- I know that the Bern Institute of Family Medicine (BIHAM) has taken out insurance. This insurance pays out if I suffer damage - but only if the damage is directly related to the study.

| Place, date | Surname and first name / participant block capitals  Signature / Participant |
| --- | --- |

**Confirmation of the study team:** I hereby confirm that I have explained the nature, significance and scope of the study to this participant. I confirm that I will fulfil all obligations under Swiss law associated with this study. Should I learn of any aspects during the course of the study that could influence the participant's willingness to take part in the study, I will inform him/her immediately.

| Place, date | Surname and first name of the representative(s) of the study team in block capitals  Signature of the representative(s) of the study team |
| --- | --- |

### 2) Declaration of consent for further use and / or disclosure of data in encrypted or anonymised form

This consent does not concern you in the sense of personal participation in a study.

"Further use" means that data can be stored beyond the time of your participation in the study and used in encrypted or anonymised form for further research.

"Disclosure" means that your data may be passed on to other research persons or research institutions in encrypted or anonymised form for further research projects. These other research persons or research institutions may also be located abroad. It is the responsibility of the sponsor to ensure that this country has an adequate level of data protection comparable to that in Switzerland.

| **BASEC number** | 2024-00685 |
| --- | --- |
| **Title of the study:** | The DROPIT study: A national clinical study on medication optimisation |
| **Responsible institution:**  (Sponsor with address) | Prof Dr med Sven Streit  Bern Institute of Family Medicine (BIHAM)  Mittelstrasse 43, 3012 Bern  Tel: +41 31 631 58 70 |
| **Surname and first name GP**  (Indicate in block capitals) |  |
| **Surname and first name of study participant:**  (Indicate in block capitals) |  |
| **Date of birth study participant:** |  |
| **Gender study participant:** | female  male  diverse |

- I give permission for my encrypted (pseudonymised) and, after 10 years, anonymised data from this study to be used and passed on for medical research.
- The data can be analysed in Switzerland and abroad (e.g. by external research partners) and stored in a database here or abroad. Research institutions abroad must comply with the same data protection standards that apply in Switzerland.
- I understand that my data is encrypted and that the key is stored securely.
- I voluntarily decide in favour of the further use and/or disclosure of data and can revoke this decision at any time. I will only inform my family doctor or dropit.biham@unibe.ch and do not have to justify this decision.
- If I withdraw from the decision to further use and/or pass on my data, my data collected up to this point will remain encrypted and anonymised in the database after 10 years and will no longer be used or passed on from this point onwards. Normally, all data is analysed in summary form. If, by chance, there is a result that is very important for my health, I will be contacted.

| Place, date | Surname and first name / participant block capitals  Signature / Participant |
| --- | --- |

**Confirmation of the study team:** I hereby confirm that I have explained the nature, significance and scope of the further use of data to the participant(s).

| Place, date | Surname and first name of the representative(s) of the study team in block capitals  Signature of the representative(s) of the study team |
| --- | --- |

### 3) Consent form for the recording of a possible consultation

This consent does not concern you in the sense of personal participation in a study.

| **BASEC number** | 2024-00685 |
| --- | --- |
| **Title of the study:** | The DROPIT study: A national clinical study on medication optimisation |
| **Responsible institution:**  (Sponsor with address) | Prof Dr med Sven Streit  Bern Institute of Family Medicine (BIHAM)  Mittelstrasse 43, 3012 Bern  Tel: +41 31 631 58 70 |
| **Surname and first name GP**  (Indicate in block capitals) |  |
| **Surname and first name of study participant:**  (Indicate in block capitals) |  |
| **Date of birth study participant:** |  |
| **Gender study participant:** | female  male  diverse |

- I agree that a possible meeting with my general practitioner may be recorded for quality assurance purposes

| Place, date | Surname and first name / participant block capitals  Signature / Participant |
| --- | --- |

**Confirmation by the study team:** I hereby confirm that I have explained the nature, significance, and scope of the project to the participant

| Place, date | Surname and first name of the representative(s) of the study team in block capitals  Signature of the representative(s) of the study team |
| --- | --- |

**The DROPIT study:**

**A national clinical study on medication optimisation**

Information for the legal representatives of the participants

Dear Sir or Madam,

Thank you very much for your interest in this study. We would like to inform you about our research project. The patient is incapable of judgement and therefore unable to communicate his/her own will to us independently. We would like to inform you about the DROPIT study and ask you to consider the patient's consent to participate in the project. You as a relative (if applicable: as a legal representative) can then give your consent on their behalf.

In this study, we want to investigate the participant's drug therapy.

Participation is voluntary. The following **information for the participants' legal representatives** is intended to help you make a decision. If you give your consent for participation, please inform the participant's family doctor. You will then be contacted by telephone at a later date by a representative of the study team to discuss the study with you in detail. Please keep this information sheet, which you have received from the GP, until this telephone call.

**If anything is unclear to you, please ask your family doctor or the contact person from the study team.** If, after your discussion with your contact person from the study team, you still wish to give your consent to participate in the study, please **sign the consent form** at the end of this information letter. With your signature, you confirm that you have read and understood this information and agree to the patient's participation in the study.

The information for the legal representatives of the participants and the declaration of consent consists of four parts:

Part 1 The most important facts in brief
Part 2 This is what it's all about in detail: Information on the study
Part 3 Data protection and insurance cover
Part 4 Declaration of consent

This study is organised by the Bern Institute of Family Medicine (BIHAM) at the University of Bern. This institution is called the sponsor. The sponsor is responsible for the initiation, management and financing of the study. The person responsible on the sponsor's side is Prof. Dr Sven Streit, BIHAM, University of Bern.

**In the context of this study is responsible for you:**

Name Prof Dr med Sven Streit

Address Bern Institute of Family Medicine (BIHAM),
Mittelstrasse 43, 3012 Bern

Telephone +41 31 631 58 70

e-mail [dropit.biham@unibe.ch](mailto:dropit.biham@unibe.ch)

Part 1: The most important facts in brief

Especially from phase 3 studies (IMP) or confirmatory MD studies.

# Why are we conducting this study?

Many people take medication on a regular basis. It may be that certain medications are unsuitable for treatment or that their benefits are insufficient. Taking these medications often has a negative impact on the patient's state of health and quality of life. For this reason, optimising (discontinuing, reducing or restarting) the medication often makes sense. In this study, we are investigating how medication optimisation can work better. You can find out more about the scientific background to the study in Part 2, **Chapter 4.**

# What does the patient have to do when participating?

Participants take part in this study for 12 to 15 months. At the beginning, participants will be randomly assigned to one of two groups. During this time, we will invite the participants to complete a questionnaire 5 times (every 3 months). The questionnaire can be completed either independently (or by you as a legal representative) electronically or during a telephone call with someone from the study team. Completing the questionnaire takes about 30 to 45 minutes.

# What are the benefits and risks associated with participation?

## Benefit

The results from this study will help us to improve guidelines for optimising medication. Direct benefits for the participants cannot be guaranteed.

## Risk

Any changes to the medication are made with the health interests of the participants in mind. Nevertheless, adverse effects can occur. For example, symptoms of an illness may reappear when a medication is discontinued or the dose is reduced. Or new symptoms may occur when taking a new medication or after increasing the dose of a medication currently in use. We are constantly working to minimise these risks as much as possible. In Part 2, **Chapter 6**, you will find further information on risks, side effects and side effects.

Part 2: This is what it's all about in detail: Information on the study

# The scientific background to the study

## Background: Why are we conducting this study?

In the case of drug treatment, it may be that (further) treatment with a drug is not (or no longer) medically appropriate. On the other hand, it can happen for various reasons that patients do not receive medication with a presumed greater benefit. Both scenarios can have negative consequences. It is therefore important to constantly optimise and update the use of medication for patients. The aim of this study is to test/develop a new strategy to support patients and GPs in optimising medication safely and effectively.

## Structure of the study: How do we proceed?

This clinical trial will be conducted in Switzerland from 2023 to 2028. A total of 80 GPs are taking part. This includes the participant's GP. Each GP will include approx. 5 participants; a total of 400 people will take part in the study.

In the study, the participating GPs are randomly divided into two groups. This is referred to as "randomisation":

- **Group 1 (trial group):** GPs in this group receive instructions on how to optimise the medication list.
- **Group 2 (control group):** GPs in this group receive no guidance and the participants are treated as usual.

Depending on the GP's group assignment, the participant's medication list is either optimised according to the instructions (**group 1**) or the GP looks after the participant according to the standard procedure (**group 2**), as would also be the case outside the study.

In this study, the participants and their legal representatives do not know into which group the participants and GPs have been categorised. The idea behind this is that the participants can consciously or unconsciously influence the results as little as possible, which could falsify the study results. The random assignment of the GPs to groups and the blinding of the participants should help us to be able to assess as objectively as possible at the end of the study whether or how well the optimisation of the medication actually works in this study.

## Regulations on scientific research involving human subjects

We conduct this study in accordance with Swiss law (Human Research Act, Data Protection Act). We also observe all internationally recognised guidelines. The responsible ethics committee has reviewed and authorised the study.

# Procedure of the study

## What do you have to do if the potential participant takes part in the study?

Participation in this study is voluntary and lasts between 12 and 15 months. Participants must adhere to the study schedule and all instructions given to them by the study team and/or the family doctor.

- You should inform the participant's family doctor if the participant's state of health changes, for example if they get worse or if new symptoms occur. This also applies if the study is terminated prematurely (see chapters 5.3 and 5.4). To ensure the safety of all participants, we want to track all health-related incidents. This is independent of whether or not they are attributable to the study. We therefore ask that you contact the participant's family doctor if you have any health concerns or events.
- It is necessary for the study participants to adhere to the requirements of the study. This includes 1) attending all appointments with their family doctor during the course of the study (if possible) for joint consultations (at the request of the family doctor) and 2) answering the study team's questions about medication intake, state of health and quality of life (every 3 months).

## What happens during the appointments?

During the study, the family doctor can invite the participants to visit at any time to discuss the medication.

**Overview of the study programme:**

|  | **What happens?** | **Timing** | **Location** | **Time required** |
| --- | --- | --- | --- | --- |
| **1** | Enquiry by GP, if interested you will receive the participant information for legal representatives and the GP will pass on your contact details to the study team | today | GP practice | 20 minutes |
| **2** | Telephone call by the study team and explanation of the details of participation, signing of the consent form at the end of this document (study inclusion) and recording of an emergency contact. | approx. 1-2 weeks before study inclusion | at home | 30-45 minutes |
| **3** | Collection of baseline data, this means filling out a questionnaire (online or with the study team). | - | at home | 30-45 minutes |
| **4** | Possible discussion with family doctor  With consent, this meeting can be recorded. | approx. 4 weeks after study inclusion | GP practice | 30-45 minutes |
| **5** | Possible completion of a symptom diary | approx. 2 weeks after the possible consultation with the family doctor | at home | 1-3 minutes per day |
| **6** | Completion of a questionnaire (online or by telephone with the study team) about the participant's state of health, quality of life and medication | 3 months after study inclusion | at home | 30-45 minutes |
| **7** | Completion of a questionnaire (online or by telephone with the study team) | 6 months after study inclusion | at home | 30-45 minutes |
| **8** | Completion of a questionnaire (online or by telephone with the study team) | 9 months after study inclusion | at home | 30-45 minutes |
| **9** | Completion of a questionnaire (online or by telephone with the study team) | 12 months after study inclusion | at home | 30-45 minutes |
| **Total** | | | | **~4-7 hours** |

## When does participation in the study end?

In total, regular study participation lasts **between 12 and 15 months**.

However, participation can be cancelled at any time. Participants and/or their legal representatives do not have to declare the cancellation of the study. If participation is terminated earlier, please discuss this with your family doctor. Even if participation is terminated prematurely, the GP will continue to provide the participants with medical care in accordance with current standards.

We may also have to ask you and the participants to end the study prematurely. This is the case, for example, if the family doctor or the study director comes to the conclusion that further participation is no longer in the best (health) interest. It may also be the case that the entire clinical trial is terminated prematurely, meaning that participation ends prematurely for all participants.

In the event of premature discontinuation of the study, we will contact the participants one last time by telephone. The data collected up to the point of discontinuation will still be analysed for the study.

## What happens if you do not wish to give your consent to participate in the study?

Even if you do not give your consent to participate in this study, the patient's medical treatment will be guaranteed regardless of your decision, and the previous treatment will continue unchanged.

# Risks, burdens and side effects

As with any medical treatment, there are risks and burdens associated with participating in this study. Some risks are already known, others are still unknown. This uncertainty is not unusual in a trial environment. For example, symptoms of a disease may reappear when a drug is discontinued or the dose is reduced. Or new symptoms may occur when taking a new drug or after increasing the dose of a drug currently in use. We are constantly working to minimise these risks and will inform you of any new findings on risks and side effects during the study. **Overall, the risks do not differ from the usual treatment. All treatment decisions are made by the participants together with their family doctor.**

For participants who might have children:

Participants may have children during and immediately after the end of the study. This applies to both women and men. Participants should discuss these questions with their investigator

# Alternative

If you decide that the patient should not take part in the study, treatment will continue as before.

# Results from the study

The overall results of the study, which come from the data of all participants, do not directly affect the health of the participants. If you provide the study team with your e-mail address, we will be happy to send you a summary of the overall results at the end of the study.

Part 3: Data protection and insurance cover

# Data protection

We protect the data of participants. There are strict legal regulations on data protection in Switzerland.

The Swiss Data Protection Act gives you the right to information, correction and receipt of the participants' data that is collected, processed and forwarded as part of the study. These rights cannot always be guaranteed in exceptional cases due to other legal or regulatory requirements. If you have any questions, please contact the investigator.

## Encryption of data

Each study generates data from the investigations. This data is documented. This is usually done electronically in large tables, the so-called "data collection sheets". In this study, these are primarily data on the medication, health status and quality of life of the participants. All data is documented in encrypted form. "Encrypted" means that personal information that could directly identify the participants is stored separately from the study results. For this purpose, there is a list (key list) that identifies each person with a unique code. Access to the key of this code is restricted and only accessible to authorised study team members. The participant's GP is aware of this code, but does not have access to the data in the database. The key list will remain with us at the institution for a period of 10 years and will be kept under the direct responsibility of the study director, after which it will be destroyed. No one else receives this key list. Special exceptions are regulated in chapter 9.4.

The personal data that we need for communication with the participants or with you (e.g. by post and telephone) is stored separately from the data in the study database. This data will be deleted immediately after the end of the program. Only the e-mail address will be stored in the database for sending the questionnaires. As soon as the study is completed or the participants wish to withdraw from the study, the e-mail address will be deleted and will no longer be included in the database.

At the end of the legally prescribed 10-year retention period, the study data will be completely anonymised. This means that it will no longer be possible to link the data to the participants without disproportionate effort. Among other things, the key list will be destroyed for anonymisation.

## Data protection

All data protection regulations are strictly adhered to. It is possible that the data must be transmitted in encrypted form, for example for publication, and can be made available to other researchers. If health-related data is stored on site, it is a database for research purposes. These data may be sent in encrypted form to another database as part of this study (specify institution, location, country, duration of storage). The sponsor is responsible for ensuring that the same standards are observed abroad as in Switzerland. However, if the same level of data protection cannot be guaranteed abroad as in Switzerland, the sponsor has taken the necessary measures to protect the rights of the participants.

**During the study:** The sponsor is responsible for the secure handling of the participants' data from the study and is responsible for ensuring that the applicable laws, e.g. data protection laws, are complied with. It is often important that the participant's general practitioner shares the participant's medical history data with the study team. By giving your consent at the end of the document, you are authorising this.

**After the study:** The sponsor remains responsible for the secure handling of your data even after the end of the study. The law stipulates that all study documents, including the data in the study database, must be kept for at least 10 years.

At the end of this long period, study data remains encrypted. Or: measures are taken to prevent the data from being assigned to you personally as far as possible. Health-relevant data from your medical history, including from this study, are and will always remain accessible to those treating you. Once a study has been completed, the results are usually published in scientific journals. The results are also scrutinised by other experts. Your coded data must be passed on to these specialists. However, the data may not be used for new research purposes. This would require your separate consent (🡪 Chapter 9.3).

## Data protection for further use

The data of the participants from this study are also very important for future research projects. Data that has already been used, as well as data that has not yet been used for this study, can possibly be used anonymised for other research projects and / or passed on (also abroad).

We require your separate consent for the further use and/or forwarding of participants' data. This is voluntary. Please read the additional declaration of consent at the end of the document carefully. Please sign the consent form if you would like to support further research projects in the future with the data of the participant(s). Even if you do not consent to the further use and/or disclosure, the participant can still take part in the study.

## Inspection rights during inspections

The conduct of this study can be reviewed. This is done by authorities such as the responsible ethics committee. The sponsor must also carry out such reviews to ensure the quality of the study and the results.

A small number of specially trained people are given access to the participants' personal data and medical history. The data is therefore not encrypted for this review. The people who see the unencrypted data are subject to a duty of confidentiality.

All study participants have the right to view their data at any time.

# Resignation

The patient can withdraw from the study at any time and terminate participation if he/she wishes to do so or if you as a relative (if applicable: as legal representative) decide to do so. The e-mail address for the automatic sending of the questionnaires will be deleted from the database on the date of withdrawal, but the data collected up to that point will still be analysed in encrypted form in this case. After a statutory period of 10 years, all data will be anonymised. This is primarily for medical safety reasons. Please check whether you agree to this before you give your proxy consent to the patient's participation in the study. After evaluation, the data will be anonymised. The key assignment will be destroyed so that no-one will be able to find out who originally provided the data. This is primarily for data protection purposes.

# Compensation

The participants' appointments with the family doctor will be billed in the usual way via the health insurance company. For an appointment with the family doctor, which is solely about the study, the family doctors will receive compensation from the sponsor and the health insurance company will not be charged anything. Participation in the study does not result in any additional costs for the participants or their health insurance companies.

# Participants will not be financially compensated for taking part in the study. Insurance cover / Liability

Participants are insured if they suffer damage as a result of taking part in the study. The procedure is regulated by law. The sponsor has taken out insurance with Baloise for this purpose. If you believe that the participant has suffered damage as a result of the study, please contact the family doctor or the insurance company directly.

Baloise Insurance Ltd
Aeschengraben 21
P.O. Box 2275
CH-4002 Basel

# Financing the study

This study is organised by the Bern Institute of Family Medicine (BIHAM) and its partners (Institute of Family Medicine Zurich, Institute of Psychology of the University of Bern, Institute of Social and Preventive Medicine Bern, Patient Service Center Basel and Zurich, Swiss Patient Safety Foundation). The study is financed by the Swiss National Science Foundation as part of the "Investigator Initiated Clinical Trials" program. The researchers involved have no direct financial advantage from conducting this study.

# Contact person

You may ask questions about the studies at any time. Please also contact us if you have any uncertainties or emergencies that arise during or after the study:

Name Prof Dr med Sven Streit

Address Bern Institute of Family Medicine (BIHAM),
Mittelstrasse 43, 3012 Bern

Telephone +41 31 631 58 70

e-mail [dropit.biham@unibe.ch](mailto:dropit.biham@unibe.ch)

Part 4: Declaration of consent

Please read this form carefully. Please ask us if anything seems unclear or if there is anything else you would like to know. This consent consists of three independent declarations of consent:

1. Consent to participation in the DROPIT study by the legal representative
2. Consent for the further use of data from this study in encrypted form, e.g. for new research purposes.
3. Consent to record a possible meeting for quality assurance.

### Declaration of consent for participation in the DROPIT study by the legal representative.

| **BASEC number** | 2024-00685 |
| --- | --- |
| **Title of the study:** | The DROPIT study: A national clinical study on medication optimisation |
| **Responsible institution:**  (Sponsor with address) | Prof Dr med Sven Streit  Bern Institute of Family Medicine (BIHAM)  Mittelstrasse 43, 3012 Bern  Tel: +41 31 631 58 70 |
| **Surname and first name GP**  (Indicate in block capitals) |  |
| **Surname and first name of study participant:**  (Indicate in block capitals) |  |
| **Surname and first name of the legal representative(s):** (Indicate in block capitals) |  |
| **Date of birth study participant:** |  |
| **Gender study participant:** | female  male  diverse |

- As the legal representative of the participant(s), I have received verbal and written information about the study from the study team.
- The study team explained the purpose, procedure and risks of the study to me.
- I confirm that I am making a decision in the interests of the person I represent, namely that they participate in this research project. I accept the verbal and written information on behalf of the person I represent. I have had sufficient time to make this decision.
- I hereby confirm that participation is voluntary for the person I represent.
- The study team explained to me what standard treatments are available outside of the study.
- The questions in connection with participation in this study have been answered. I will keep the written information and receive a copy of the written declaration of consent.
- I agree that the family doctor will be informed about my participation in the study.
- I have had sufficient time to make this decision. I will keep the written information and receive a copy of the written declaration of consent.
- I can withdraw the participation of the participant I represent at any time. I do not have to explain why. Even if participation has ended, the person I represent will continue to receive medical treatment. The data collected up to that point will remain stored and will be analysed as part of the study. The data will remain encrypted. The contact details from the database will be deleted and no further data of the participant will be collected.
- I am aware that the obligations stated in the information leaflet must be complied with. In the interest of health, the investigator may exclude the patient at any time.
- The responsible specialists of the sponsor and the ethics committee may inspect the unencrypted data of the person I represent for control purposes. All these persons are subject to a duty of confidentiality.
- I know that the Bern Institute of Family Medicine (BIHAM) has taken out insurance. This insurance pays out if the participant suffers an injury - but only if the injury is directly related to the study.

**Confirmation of the next of kin, legal representative:** I/we hereby confirm that the informed consent discussion has taken place and that the person lacking capacity has consented to participate in the study and/or that there are no signs of resistance to participation.

| Place, date | Surname and first name / legal representative of the participant in capital letters:  Details of the relationship with the patient  Signature / legal representative of the participant |
| --- | --- |

**Confirmation of the study team:** I hereby confirm that I have explained the nature, significance and scope of the project to the person signing above on behalf of the participant. I confirm that I will fulfil all obligations in connection with this research project in accordance with applicable law. If at any time during the execution of the research project I become aware of aspects that could influence the participant's willingness to take part in the research project, I will inform the person on my behalf immediately.

| Place, date | Surname and first name of the representative(s) of the study team in block capitals  Signature of the representative(s) of the study team |
| --- | --- |

### 2) Declaration of consent for further use and/or disclosure of data in encrypted or anonymised form

This consent does not concern the person I represent in the sense of personal participation in a study.

"Further use" means that data can be stored beyond the period of study participation and used in encrypted or anonymised form for further research.

"Passing on" means that the data may be passed on to other research persons or research institutions in encrypted or anonymised form for further research projects. These other research persons or research institutions may also be located abroad. It is the responsibility of the sponsor to ensure that this country has an appropriate level of data protection comparable to that in Switzerland.

| **BASEC number** | 2024-00685 |
| --- | --- |
| **Title of the study:** | The DROPIT study: A national clinical study on medication optimisation |
| **Responsible institution:**  (Sponsor with address) | Prof Dr med Sven Streit  Bern Institute of Family Medicine (BIHAM)  Mittelstrasse 43, 3012 Bern  Tel: +41 31 631 58 70 |
| **Surname and first name GP**  (Indicate in block capitals) |  |
| **Surname and first name of study participant:**  (Indicate in block capitals) |  |
| **Surname and first name of the legal representative(s):** (Indicate in block capitals) |  |
| **Date of birth study participant:** |  |
| **Gender study participant:** | female  male  diverse |

- I authorise that the encrypted (pseudonymised) and after 10 years also anonymised data of the person I represent from this study may be further used and passed on for medical research.
- The data can be analysed in Switzerland and abroad (e.g. by external research partners) and stored in a database here or abroad. Research institutions abroad must comply with the same data protection standards that apply in Switzerland.
- I understand that the data of the person I represent is encrypted and the key is stored securely.
- I understand that this decision is voluntary and I can withdraw this decision at any time. I will only inform the participating person's family doctor or dropit.biham@unibe.ch and do not have to justify this decision.
- If I withdraw from the decision to further use and/or pass on the data of the person I represent, the data collected up to this point will remain encrypted and anonymised in the database after 10 years and will no longer be used or passed on from this point onwards. Normally, all data is analysed in aggregate. If, by chance, a result emerges that is very important for the health of the person I represent, I will be contacted.

**Confirmation of the next of kin, the legal representative:**

I/we hereby confirm that the informed consent discussion has taken place and that the person lacking capacity has consented to the further use of data in encrypted or anonymised form and/or that there are no signs of resistance to participation.

| Place, date | Surname and first name / legal representative(s) of the participant block capitals (if applicable)  Details of the relationship with the patient  Signature / legal representative of the participant(s) |
| --- | --- |

**Confirmation of the study team:** I hereby confirm that I have explained the nature, significance and scope of the project to the person signing above on behalf of the participant

| Place, date | Surname and first name of the representative(s) of the study team in block capitals  Signature of the representative(s) of the study team |
| --- | --- |

### 3) Consent form for the recording of a possible consultation

This consent does not concern you in the sense of personal participation in a study.

| **BASEC number** | 2024-00685 |
| --- | --- |
| **Title of the study:** | The DROPIT study: A national clinical study on medication optimisation |
| **Responsible institution:**  (Sponsor with address) | Prof Dr med Sven Streit  Bern Institute of Family Medicine (BIHAM)  Mittelstrasse 43, 3012 Bern  Tel: +41 31 631 58 70 |
| **Surname and first name GP**  (Indicate in block capitals) |  |
| **Surname and first name of study participant:**  (Indicate in block capitals) |  |
| **Surname and first name of the legal representative(s):** (Indicate in block capitals) |  |
| **Date of birth study participant:** |  |
| **Gender study participant:** | female  male  diverse |

- I agree that a possible meeting with the general practitioner may be recorded for quality assurance purposes

**Confirmation by relatives or legal representatives**: I/we hereby confirm that the informational discussion has taken place and that the person I/we represent has consented to the recording and/or that no signs of resistance to the recording are evident.

| Place, date | Surname and first name / legal representative(s) of the participant block capitals (if applicable)  Details of the relationship with the patient  Signature / legal representative of the participant(s) |
| --- | --- |

**Confirmation by the study team:** I hereby confirm that I have explained the nature, significance, and scope of the project to the above-signed person acting on behalf of the participant.

| Place, date | Surname and first name of the representative(s) of the study team in block capitals  Signature of the representative(s) of the study team |
| --- | --- |
