# Supplementary material for: DepRescribing inapprOpriate Proton Pump InhibiTors (DROPIT): study protocol of a cluster-randomised controlled trial in Swiss primary care
Source: BMJ Open. 2025 Jan 20;15(1):e094495. doi: 10.1136/bmjopen-2024-094495 (PMC11749314; doi:10.1136/bmjopen-2024-094495)
Supplement: Supplementary file 3 [file bmjopen-2024-094495.reviewer_comments.pdf]

## PEER REVIEW HISTORY

BMJ Open publishes all reviews undertaken for accepted manuscripts. Reviewers are asked to complete a checklist review form and are provided with free text boxes to elaborate on their assessment. These free text comments are reproduced below.

### ARTICLE DETAILS

**Title (Provisional)**

DepRescribing inapprOpriate Proton Pump InhibiTors (DROPIT): study protocol of a cluster-randomised controlled trial in Swiss primary care

**Authors**

Schulthess-Lisibach, Angela Edith; Lüthold, Renata Vidonscky; Tombez, Clémentine; Weir, Kristie; Zangger, Martina; Chan, Samantha; Jenal, Flurina; Roumet, Marie; Mattmann, Yvonne; Bieri, Christof; Aubert, Carole Elodie; Rodondi, Nicolas; Zambrano Ramos, Sofia Carolina; Trelle, Sven; Neuner-Jehle, Stefan; Juillerat, Pascal; Barbier, Michaela Carla; Inauen, Jennifer; Streit, Sven; Jungo, Katharina Tabea; Vallejo-Yagüe, Enriqueta

---

### VERSION 1 - REVIEW

---

| | |
|---|---|
| **Reviewer** | **1** |
| **Name** | **Haastrup, Peter** |
| **Affiliation** | **Research Unit of General Practice, University of Southern Denmark** |
| **Date** | **15-Oct-2024** |
| **COI** | **None** |

---

Thank you for the invitation to review this manuscript that is a study protocol for an RCT in Swiss primary care regarding deprescribing of PPI.

Inappropriate use of PPI is continously important to address and interventions in primary care aimed at improving rational prescribing are warranted so I find the paper very relevant. The manuscript is well written and the methodology sound.

I only have few suggestions for the authors to consider to increase the transparency of the study and the in-depth aspects of PPI prescribing and how this study fits into the current knowledge:

Knowledge about the views and thoughts of GPs and patients regarding PPI prescribing might be relevant for the authors to consider in the understanding of possible

barriers/facilitators for PPI deprescribing, e.g. Thompson et al BMC Fam Pract. 2020 Aug 8;21(1):160

The study seems like a rather complex intervention. Have you considered using the MRC framework for complex interventions (Skivington et al BMJ 2021; 374 doi: https://doi.org/10.1136/bmj.n2061) or other guidelines for the development and evaluation of complex interventions?

You describe the patient recruitment procedure well but how will the GPs be recruited?

Which effect (if any) do you think that participation as a GP assigned to the control group will have on their prescribing behavior? Is it possible that GPs volunterring to participate in a deprescribing trial will be more attentive towards rational prescribing of PPI despite being allocated to the control group?

Have you considered efforts/information to the GPs so that GPs in the intervention arm do not share the intervention material with GPs in the usual care arm hampering your randomisation?

You aim at recruiting 400 patients but need 360 patients for the safety endpoints according to your power calculation. Can you qualify that no more than a 10% drop-out rate is realistic for a rather extensive long-term follow-up study?

What is the reminder procedure if a patient fails to respond to the surveys? Could you describe this in more detail?

The ethics section is very short for an intervention study. Could you elaborate on the ethical aspects?

---


| | |
|---|---|
| **Reviewer** | **2** |
| **Name** | **Shakib, Sepehr** |
| **Affiliation** | **University of Adelaide, Discipline of Pharmacology** |

| | |
|---|---|
| **Date** | **21-Oct-2024** |
| **COI** | **None** |

Patient selection in these studies is very important hence I would appreciate further clarification around the exclusion criteria of "antiplatelet therapy" and "additional antiplatelet therapy" and whether these include low dose aspirin or not, and whether if the patient is on clopidogrel and ticagrelor whether this also counts as 2 antiplatelets. Also further clarification on what type of severe concomitant disease would be exclusionary would be appreciated.

Further clarification of the process of recruitment of participants in this trial would be appreciated, in particular how the patients will be identified and consented. Will there be a process of identifying potential patients from a patient database and having them approached for the study by an investigator other than the GP, or will the expectation be that the GPs will enroll the patients as they see them for their usual process of care? The issue here is that patients who are inappropriately on PPIs are unlikely to be seeing their GPs about this issue, and the GPs are likely to have to opportunistically enroll patients into this study e.g. when they present for other reasons. In this scenario, then one can expect many more patients to be recruited in the intervention arm, as the control GPs do not have anything to offer the patients. The patients in the control arm are likely to represent a biased subset of patients who present with inappropriate PPI prescribing. The incentives offered to GPs to enroll control patients is also an important consideration to elaborate on, if possible.

In terms of the additional safety measures, is there a systematic process in place to identify these endpoints such as B12, iron deficiency in the participants, or will this be up to voluntary reporting by the GPs?

## VERSION 1 - AUTHOR RESPONSE

| **Reviewer 1, Dr. Peter Haastrup, Research Unit of General Practice:** | |
|---|---|
| Thank you for the invitation to review this manuscript that is a study protocol for an RCT in Swiss primary care regarding deprescribing of PPI.<br>Inappropriate use of PPI is continuously important to address and interventions in primary care aimed at improving rational prescribing are warranted so I find the paper very relevant. The manuscript is well written and the methodology sound.<br>I only have few suggestions for the authors to consider to increase the transparency of the study and the in-depth aspects of PPI | Thank you for this general appraisal of the manuscript and your valuable comments. Below we address each comment individually. |

| | |
|---|---|
| prescribing and how this study fits into the current knowledge: | |
| Knowledge about the views and thoughts of GPs and patients regarding PPI prescribing might be relevant for the authors to consider in the understanding of possible barriers/facilitators for PPI deprescribing, e.g. Thompson et al BMC Fam Pract. 2020 Aug 8;21(1):160 | Thank you for this comment. We have incorporated the views and thoughts from GPs and patients in the development of our intervention. We plan to explain more about this in a different manuscript focused on the deprescribing intervention development that will be published separately. We thus think that this level of detail about the intervention development is not desired for this manuscript. Yet, we acknowledge the importance of mentioning that this was taken into consideration. Here we highlight the sentence:<br><br>**Page 9:**<br>**'…It was developed to address the barriers and enablers of PPI deprescribing at patient and GP levels, and the materials were co-designed and adapted based on patient and GP feedback.'** |
| The study seems like a rather complex intervention. Have you considered using the MRC framework for complex interventions (Skivington et al BMJ 2021; 374 doi: https://doi.org/10.1136/bmj.n2061) or other guidelines for the development and evaluation of complex interventions? | The development and evaluation of this complex intervention was informed by the MRC framework for complex interventions (Skivington et al., 2022), the person-based approach (Yardley et al., 2015; https://doi.org/10.2196/jmir.4055), and the behavior change wheel (Michie et al., 2014; *The behaviour change wheel. A guide to designing interventions*. Silverback Publishing.). The combination of these approaches have guided us to develop an intervention based on available theory, evidence, and by co-designing the intervention together with stakeholders for contextualization to the Swiss primary care setting. The details of this approach will be published in a separate article. For the evaluation, besides the efficacy analysis, we incorporate analyses of fidelity, dose-response, mechanisms of intervention as well as cost-effectiveness. |
| You describe the patient recruitment procedure well but how will the GPs be recruited? | The GPs participating in our study are not considered study participants. Therefore, we decided not to talk about the recruitment of GPs, to avoid misunderstandings. However, answering the comment from the reviewer, we engage GPs through various channels such as presenting the study in quality circles (i.e., educational meetings for GPs), distributing flyers and emails via GP networks such as the mediX group and personal contacts. We added a sentence in the strength and limitation section.<br><br>**Page 21:** |

| | |
|---|---|
| | **‘Furthermore, GPs are encouraged to join the trial through various channels such as quality circles (i.e., educational meetings for GPs), distributing flyers and emails via GP networks or personal contacts. Although, the network is widely spread within the German-speaking part of Switzerland, it does not fully represent the entire GP population since the French- and Italian-speaking parts are not covered.’** |
| Which effect (if any) do you think that participation as a GP assigned to the control group will have on their prescribing behavior? Is it possible that GPs volunteering to participate in a deprescribing trial will be more attentive towards rational prescribing of PPI despite being allocated to the control group? | We share the concern you have raised. However, these phenomena would be apparent in any study that focuses on optimization of medication lists, and we cannot change this. In a pilot study by Lüthold et al. from our group, we observed that despite identifying inappropriate PPI prescriptions, over 60% of cases saw no change in the following 12 months. This was often due to reluctance to discontinue the medication, fear of recurring symptoms, or lack of patient-provider discussions. Therefore, we think simple awareness through study participation will not automatically result in a different prescription pattern.<br>To address this comment, we added a paragraph to the limitation section.<br><br>**Page 20:**<br>**‘One could hypothesize that the GPs participating in a deprescribing study may be more attentive to reducing inappropriate prescribing. However, in the study by Lüthold et al. from our team, we observed that when GPs were instructed to actively identify inappropriate PPIs in patient records, only 35% of the inappropriate PPIs were stopped or reduced after 12 months, while over 60% remained unchanged. [24] Reasons for a failed deprescribing included lack of discussion with patients, their unwillingness to stop, and fear of recurrence of symptoms. Therefore, it cannot be excluded that an increased awareness among control group patients will bias the trial results towards the null, but the effect is likely to be small.’** |
| Have you considered efforts/information to the GPs so that GPs in the intervention arm do not share the intervention material with GPs in the usual care arm hampering your randomisation? | The reviewer rises a very good point, and we understand the concern. While we did consider to cluster on practice level, there were two main reasons that support clustering by GP: (1) achieving more similar sample sizes across clusters and (2) aiding with the challenge of recruiting GPs for the trial, facilitating direct one-to-one contact. Clustering at the GP level maximizes the number of clusters with balanced |

| | |
|---|---|
| | sample sizes. In contrast, clustering by practice would result in diverse cluster sizes and complicate recruiting enough patients. Our approach leverages positive experiences from trial GPs, who may encourage peers to join, thereby creating new clusters and increasing patient participation.<br><br>In DROPIT, we explicitly informed the GPs to not talk about the study with colleagues and to not share or distribute any material we give to them. This might be a concern in a practice with several GPs from the trial, potentially assigned to different trial arms. However, GPs generally respect confidentiality and have limited time to discuss study participation due to their busy schedules. Patients likely continue seeing their original GP (who recruited them) and, when consulting a different GP, it is expected that this consultation addresses immediate concerns rather than reviewing all medication records.<br>This issue has been addressed in the limitation section and was reformulated to address the reviewer's comment:<br><br>**Page 21:**<br>**'Each GP is treated as a separate cluster and GPs are requested to keep confidentiality with colleagues. This is particularly relevant when several trial GPs work in the same practice. Otherwise, a contamination across GP clusters may potentially bias the trial results toward the null hypothesis, underestimating the intervention effect. However, GPs are used to maintaining confidentiality among colleagues and have little time to discuss the trial with peers, thus, we trust that group contamination will be minimal.'** |
| You aim at recruiting 400 patients but need 360 patients for the safety endpoints according to your power calculation. Can you qualify that no more than a 10% drop-out rate is realistic for a rather extensive long-term follow-up study? | The expected maximum 10% drop-out rate is based on the team former experience in clinical trials in similar setting (e.g. OPTICA trial by Jungo et al. Optimising prescribing in older adults with multimorbidity and polypharmacy in primary care (OPTICA): cluster randomised clinical trial \| The BMJ) |
| What is the reminder procedure if a patient fails to respond to the surveys? Could you describe this in more detail? | We have set up automatic reminder emails that are sent out every two weeks. For participants who prefer to be contacted by phone, we track our interaction with them in a separate spreadsheet where we schedule reminder calls in the same time frames as the automatically released reminder emails. |

| | |
|---|---|
| The ethics section is very short for an intervention study. Could you elaborate on the ethical aspects? | We agree and elaborate this section with more detail and it reads now as follows:<br><br>**Page 23:**<br>**'We greatly value and respect the contributions of those who share their clinical data and insights from practice with us. We are fully committed to upholding their rights, which include ensuring privacy, obtaining informed consent (supplementary B), and respecting their right to withdraw from the study at any time. GPs who recruit patients are financially compensated for their time and expertise as healthcare professionals involved in the study. While many patients join the study out of a desire to contribute to research, we also expect that, as users of PPI medication, they may experience direct benefits from participating. The risks in this study are minimal, as deprescribing is part of routine clinical practice, and the study intervention does not require any measures beyond this. Resuming medication is possible at any time during the study, and participants can withdraw from the study at any time. Additionally, in our efforts to create social value, we actively involve a range of stakeholders in our decision-making processes to ensure that diverse perspectives are considered (see the section on 'patient and public involvement'). The study will be conducted in accordance with principles in the current version of the Declaration of Helsinki, [50] the relevant parts of the guidelines of Good Clinical Practice (GCP) from the International Council for Harmonisation (ICH), as well as local regulations. This study has been approved by the Ethics Committee of Bern, Switzerland (BASEC ID: 2024-00685).'** |
| **Reviewer 2, Dr. Sepehr Shakib, University of Adelaide, Royal Adelaide Hospital:** | |
| Patient selection in these studies is very important hence I would appreciate further clarification around the exclusion criteria of "antiplatelet therapy" and "additional antiplatelet therapy" and whether these include low dose aspirin or not, and whether if the patient is on clopidogrel and ticagrelor whether this also counts as 2 antiplatelets. Also further clarification on what type of severe concomitant disease would be exclusionary would be appreciated. | Thank you for this comment. Regarding the exclusion criteria 'antiplatelet therapy' and 'additional antiplatelet therapy': yes, low dose aspirin, e.g. long-term use after myocardial infarction, is included in these two criteria.<br>If a patient is taking low-dose aspirin and clopidogrel, low-dose aspirin would be considered as 'antiplatelet therapy' and clopidogrel would be considered as 'additional antiplatelet therapy'. Hence the patient would fulfill two of the medication exclusion criteria and thus be excluded. |

| | |
|---|---|
| | If a patient is taking low-dose aspirin only and takes no other medication or has no risk factor listed in the exclusion criteria, the patient would be included.<br>The 'severe concomitant disease' that is listed in the risk factors is according to the GPs assessment (important!) and could be severe liver disease, neoplasia or severe nicotine or alcohol misuse etc. To address this comment we added the examples in the exclusion criteria.<br><br>**Page 8:**<br>**'…severe concomitant disease with increased risk of gastrointestinal bleeding according to the GP's assessment (i.e. severe liver disease, neoplasia, severe misuse of alcohol and nicotine).'** |
| Further clarification of the process of recruitment of participants in this trial would be appreciated, in particular how the patients will be identified and consented. Will there be a process of identifying potential patients from a patient database and having them approached for the study by an investigator other than the GP, or will the expectation be that the GPs will enroll the patients as they see them for their usual process of care? The issue here is that patients who are inappropriately on PPIs are unlikely to be seeing their GPs about this issue, and the GPs are likely to have to opportunistically enroll patients into this study e.g. when they present for other reasons. In this scenario, then one can expect many more patients to be recruited in the intervention arm, as the control GPs do not have anything to offer the patients. The patients in the control arm are likely to represent a biased subset of patients who present with inappropriate PPI prescribing. The incentives offered to GPs to enroll control patients is also an important consideration to elaborate on, if possible. | We gladly clarify the process of recruitment of participants.<br>Once a GP decides to enroll in the DROPIT study, they have two options for recruiting potential participants: (1) Screening medical records to identify eligible patients and inviting them to a consultation or (2) Recruiting eligible patients during routine care visits as part of their usual clinical practice. When a potential participant visits the practice, the GP will shortly summarize the study and hand them a printed informed consent form, which includes more details about the study. With the patient's consent, the GP will share their contact information with the study team. The study team will follow up by phone to guide the participant through the informed consent process, address any questions, and ensure understanding. If the participant agrees to take part, they will sign the informed consent form and send it to the study team. Once received, the PI will sign the form too, and a copy will be returned to the participant for their records. Each GP is expected to recruit between 2 and 12 participants and will be compensated with 200 Swiss francs per patient. Please note that due to limitations in the Swiss healthcare system, potential participants cannot be identified directly through electronic health records or patient databases by study team members.<br>Finally, randomisation and allocation to the intervention or the control group happens after patient recruitment is complete and the GP cluster is closed (i.e., no more participants recruited by that GP). The start of follow-up is 15 days after randomization. |

| | |
|---|---|
| In terms of the additional safety measures, is there a systematic process in place to identify these endpoints such as B12, iron deficiency in the participants, or will this be up to voluntary reporting by the GPs? | We collect diagnosis data from the GPs at every follow-up timepoint. E.g. we ask, ‘Did the patient have a vitamin B12 deficiency since the last time point we collected data?’. These are all mandatory fields and need to be answered to save and submit the survey. |

## VERSION 2 - REVIEW


**Reviewer** 1

**Name** Haastrup, Peter

**Affiliation** Research Unit of General Practice, University of Southern Denmark


**Date** 17-Dec-2024

**COI**

Thank you for your responses to my comments. I think the authors have answered my comments satisfactorily and I recommend that the manuscript is accepted for publication.
